# Photosynthetic capacity is reduced by warming but unaffected by elevated CO_2_ in seedlings of five boreal tree species

**DOI:** 10.1093/plphys/kiaf380

**Published:** 2025-08-29

**Authors:** Julia M Hammer, Mirindi Eric Dusenge, Nick Bither, Andrew Cook, André G Duarte, Kiana Lee, Bridget K Murphy, Melissa A Pastore, Stephanie C Schmiege, Robyn Swartman, Raimundo Bermudez, Norman P A Hüner, Peter B Reich, Danielle A Way

**Affiliations:** Department of Biology, University of Western Ontario, London, ON, Canada, N6A 3K7; Department of Biology, University of Western Ontario, London, ON, Canada, N6A 3K7; Department of Geography, Western Centre for Climate Change, Sustainable Livelihoods and Health, University of Western Ontario, London, ON, Canada, N6A 3K7; Department of Biology, Mount Allison University, Sackville, NB, Canada, E4L 1E2; Research School of Biology, Australian National University, Acton, ACT 2600, Australia; Department of Forest Resources, University of Minnesota, Saint Paul, MN 55108, USA; Department of Biological Sciences, University of Denver, Denver, CO 80208, USA; Department of Biology, University of Western Ontario, London, ON, Canada, N6A 3K7; Department of Biology, University of Western Ontario, London, ON, Canada, N6A 3K7; Department of Biology, University of Western Ontario, London, ON, Canada, N6A 3K7; Department of Biology, University of Western Ontario, London, ON, Canada, N6A 3K7; Department of Biology, University of Toronto Mississauga, Mississauga, ON, Canada, L5L 1C6; Graduate Program in Cell and Systems Biology, University of Toronto, Toronto, ON, Canada, M5S 3G3; Department of Ecology, Evolution, and Behavior, University of Minnesota, Saint Paul, MN 55108, USA; USDA Forest Service, Northern Research Station, St. Paul, MN 55108, USA; Department of Biology, University of Western Ontario, London, ON, Canada, N6A 3K7; Plant Resilience Institute, Michigan State University, East Lansing, MI 48824, USA; Department of Biology, University of Western Ontario, London, ON, Canada, N6A 3K7; Department of Forest Resources, University of Minnesota, Saint Paul, MN 55108, USA; Department of Biology, University of Western Ontario, London, ON, Canada, N6A 3K7; Department of Forest Resources, University of Minnesota, Saint Paul, MN 55108, USA; Hawkesbury Institute for the Environment, Western Sydney University, Penrith, NSW 2753, Australia; Institute for Global Change Biology and School for the Environment and Sustainability, University of Michigan, Ann Arbor, MI 48109, USA; Department of Biology, University of Western Ontario, London, ON, Canada, N6A 3K7; Research School of Biology, Australian National University, Acton, ACT 2600, Australia; Environmental & Climate Sciences Department, Brookhaven National Laboratory, Upton, NY 11973, USA; Nicholas School of the Environment, Duke University, Durham, NC 27708, USA

## Abstract

Increasing atmospheric CO_2_ concentrations fuel global warming, with boreal regions warming at a faster rate than many other areas. Boreal forests are an important component of the global carbon cycle, yet we have little data on photosynthetic responses of boreal trees to elevated CO_2_ (EC) and warming. We grew seedlings of 5 widespread North American boreal tree species (from *Betula*, *Larix*, *Picea*, and *Pinus*) under current (410 ppm) or elevated (750 ppm) CO_2_ and either ambient (+0 °C) or increased (+4 °C or +8 °C) temperature, then measured photosynthetic traits over a range of leaf temperatures. Our results were generally consistent across species: photosynthetic capacity (maximum rates of Rubisco carboxylation, *V*_cmax_, and electron transport, *J*_max_) was unaffected by EC but decreased under +8 °C warming. Accordingly, net photosynthesis measured at the growth CO_2_ concentration (*A*_growth_) was reduced under warming and increased under EC. The thermal optimum for *A*_growth_ (*T*_optA_) increased by ∼1.8 °C with EC but increased with warming in only two species. In contrast, the activation energies and thermal optima for *V*_cmax_ and *J*_max_, which are used to estimate photosynthesis in Earth System Models, were unaffected by growth environment. There were a few interactions between growth, CO_2,_ and warming. These results suggest increased photosynthesis of widespread boreal tree species under EC may be offset by future reductions in photosynthetic capacity related to warming. We also show that the temperature sensitivities of parameters used to estimate global photosynthesis in large-scale models are generally unaffected by simulated climate change in these species.

## Teaser

In 5 boreal tree species grown under future climate conditions, the capacity to fix CO_2_ was strongly reduced under warmer growth temperatures, but mostly unaffected by high growth CO_2_ concentrations.

## Introduction

Over the last 170 years, human activities have increased atmospheric CO_2_ concentrations ([CO_2_]) from 260 to 420 ppm (NOAA 2023), resulting in global mean annual surface warming of 0.95–1.20 °C (Arias et al. 2021). If CO_2_ emissions continue at current rates, then atmospheric [CO_2_] could reach up to 1000 ppm by 2,100 (Canadell et al. 2021), leading to surface temperatures that are ∼4.8 °C above current levels (Lee et al. 2021). These large-scale climatic changes will have serious consequences for biological systems, especially in high latitude regions, such as the boreal region, where warming is occurring most rapidly. Boreal forests account for 30% of the world's forested area (FAO and UNEP 2020) and represent one of the largest stocks of carbon (C) on Earth (Pan et al. 2011), making their response to climate change particularly important for future global C cycling.

Plants play an important role in regulating atmospheric [CO_2_] via photosynthesis. Globally, terrestrial photosynthesis assimilates ∼123 Pg of C annually, with roughly half of this entering longer-term storage as plant biomass and soil C (Amthor and Baldocchi 2001; Beer et al. 2010). However, photosynthesis is sensitive to changes in atmospheric [CO_2_] (Drake et al. 1997) and temperature (Yamori et al. 2014), making it crucial that we understand how these climatic changes alter photosynthetic processes. Photosynthesis in C_3_ plants is quantitatively described by the Farquhar-von Caemmerer-Berry (FvCB) model (Farquhar et al. 1980; Sharkey 1985), which is used by Earth System Models (ESMs) to estimate global photosynthetic CO_2_ uptake under current and future climatic conditions. In the FvCB model, net CO_2_ assimilation rates (*A*_net_) are determined by 3 biochemical processes: the maximum rate of Rubisco carboxylation (*V*_cmax_), the maximum rate of electron transport to regenerate ribulose-1,5-bisphosphate (RuBP) (*J*_max_), and the rate of triose phosphate utilisation. Triose phosphate utilisation limits *A*_net_ under high irradiance, very high [CO_2_], and/or low temperatures and is thus largely left out of ESMs (Kumarathunge et al. 2019; Rogers et al. 2021). By contrast, both *V*_cmax_ and *J*_max_ (i.e. photosynthetic capacity) and their short- and long-term responses to [CO_2_] and temperature are important inputs for ESMs (Rogers et al. 2017; Mercado et al. 2018; Kumarathunge et al. 2019; Crous et al. 2022; Oliver et al. 2022).

Short-term (minutes to hours) increases in [CO_2_] lead to increased *A*_net_ by increasing substrate availability for Rubisco carboxylation (von Caemmerer 2000). Longer-term (days, months, and years) exposure to elevated [CO_2_] (EC) also stimulates net photosynthesis measured at the growth CO_2_ concentration (*A*_growth_), but this response is often less than predicted from short-term responses (Long et al. 2004), and even this initial stimulation may disappear entirely as plants acclimate to EC, mainly due to reductions in carbon sink strength (Körner 2006; Norby et al. 2010; Warren et al. 2015). Photosynthetic acclimation to EC is associated with reductions in concentrations of Rubisco and other photosynthetic proteins and enzymes (Moore et al. 1999; Ainsworth and Long 2005). These reductions in photosynthetic enzymes are primarily driven by decreased leaf-level demand to build and maintain them under high CO_2_ availability in the intercellular space, which increases Rubisco carboxylation rates relative to oxygenation rates (Dong et al. 2022). This shift leads to source—sink imbalances, i.e. greater sugar production than the plant can utilize, which in turn suppresses Rubisco transcription and content (Moore *et al.* 1999; Ainsworth and Rogers 2007). Overall, this lowers photosynthetic capacity but improves nitrogen use efficiency (Perkowski et al. 2025). Acclimation to EC is common, but its magnitude can vary: *V*_cmax_ was reduced by 17–18% in shrubs and grasses grown under EC, but only by 6–12% in trees and legumes exposed to EC conditions (Ainsworth and Long 2005). The decrease in *J*_max_ with elevated CO_2_ is typically less pronounced than that of *V*_cmax_, resulting in an increase in the *J*_max_/*V*_cmax_ ratio in elevated CO_2_-grown plants compared to their ambient CO_2_-grown counterparts (Ainsworth and Long 2005; Dusenge et al. 2024).

Photosynthesis has a well-established, unimodal relationship with short-term changes in temperature (Berry and Björkman 1980; Sage and Kubien 2007; Yamori et al. 2014). Because *A*_net_ is determined by CO_2_ supply, photosynthetic capacity, and respiratory CO_2_ loss, the temperature response curve of *A*_net_ is determined by the temperature responses of stomatal conductance (*g*_s_), mesophyll conductance, *V*_cmax_, *J*_max_, and respiration (Sage and Kubien 2007; Lin et al. 2012). *V*_cmax_ Increases exponentially between 0 °C and ∼35 °C, typically peaking at a thermal optimum (*T*_optV_) near 40 °C, and then rapidly declining with further warming; the thermal response of *J*_max_ is similar, but its thermal optimum (*T*_optJ_) is usually slightly lower (∼30 °C) (Medlyn et al. 2002). Both of these responses can be modelled by a peaked Arrhenius function (Medlyn et al. 2002), where the thermal optima, maximum rates, and activation energies (*i*.*e*. sensitivity to temperature below the thermal optimum) of *V*_cmax_ and *J*_max_ are important descriptive parameters.

Long-term warming can alter the short-term thermal response of *A*_net_ in several ways, including adjustments to the thermal optimum of net photosynthesis (*T*_optA_) and the maximum rate of *A*_net_ (*A*_opt_) via changes in photosynthetic biochemistry and stomatal conductance (Berry and Björkman 1980; Sage and Kubien 2007; Yamori et al. 2014; Reich et al. 2018; Kumarathunge et al. 2019; Crous et al. 2022; Stefanski et al. 2023). Long-term warming usually leads to a higher *T*_optA_, which can improve *A*_net_ at higher leaf temperatures (Way and Yamori 2014; Sendall et al. 2015). For instance, in 2 field-grown boreal tree species, *T*_optA_ increased ∼0.3 °C per 1 °C of warming, stimulating *A*_net_ in the warm-grown trees at their higher growth temperature (Dusenge et al. 2023). In other cases, however, *A*_opt_ is reduced under warming conditions regardless of changes to *T*_optA_, implying that thermal acclimation is not always fully compensatory (Way and Yamori 2014). Long-term warming can also lead to alterations in *V*_cmax_ and *J*_max_ via shifts in the basal rate (i.e. photosynthetic capacity measured at a common leaf temperature) (Way and Sage 2008a; Dusenge et al. 2020), and possibly also via changes in the thermal optima and activation energies of *V*_cmax_ and *J*_max_. While many studies see no effect of warming on basal rates of *V*_cmax_ and *J*_max_ in trees (Kattge and Knorr 2007; Way and Oren 2010; Lamba et al. 2018; Kumarathunge et al. 2019; Stefanski et al. 2020; Bermudez et al. 2021), basal rates of *V*_cmax_ and *J*_max_ sometimes decrease with warming in greenhouse-grown boreal seedlings (Way and Sage 2008a, 2008b; Dusenge et al. 2020; but see Way and Yamori 2014; Murphy and Way 2021; Crous et al. 2022). Increases in *T*_optV_ and *T*_optJ_ with higher growth temperatures are more consistent in the literature (Kattge and Knorr 2007; Way and Yamori 2014; Yamori et al. 2014; Kumarathunge et al. 2019; Crous et al. 2022), including among boreal trees (Dusenge et al. 2020, 2023). By contrast, evidence for shifts in the activation energies of photosynthetic capacity (*E*_aV_ and *E*_aJ_) is mixed (Hikosaka et al. 2006; Kattge and Knorr 2007).

Elevated [CO_2_] can either offset or enhance the effects of long-term warming on photosynthesis. For example, CO_2_ loss via photorespiration is enhanced by warming but reduced by EC (Long 1991; Wujeska-Klause et al. 2019), resulting in higher *T*_optA_ in CO_2_-enriched plants when measured at prevailing growth [CO_2_] (Sage and Kubien 2007). Alternatively, low stomatal conductance under EC may exacerbate the effects of warming (Ainsworth and Long 2005) by improving plant water savings but limiting evaporative cooling (Long et al. 2004). In the latter case, *A*_net_ is doubly reduced by low intercellular [CO_2_] and high photorespiration, and may be further suppressed by heat damage (e.g. Warren et al. 2011). Despite their importance for ESMs, the temperature sensitivities of *V*_cmax_ and *J*_max_ (i.e. *T*_optV_, *T*_optJ_, *E*_aV_, and *E*_aJ_) are rarely measured in CO_2_× warming studies, such that we cannot predict how these two global change factors will affect the thermal response of photosynthetic capacity. One exception is Dusenge et al. (2020), who showed that photosynthetic capacity was reduced under warming conditions but unaffected by growth at elevated [CO_2_] in two boreal conifers, resulting in high *A*_net_ in CO_2_-enriched plants and low rates in warm-grown plants, with no CO_2_× warming interactions.

Boreal forests play an important role in regulating global C cycling, and although boreal trees are considered temperature- and nutrient-limited, there are only a few reports of boreal tree responses to EC and warming. Evergreen conifers are often considered less responsive to EC than their deciduous, broad-leaved counterparts (Medlyn et al. 2001), although slower-growing and longer-lived conifers can respond more strongly to EC than deciduous trees (Tjoelker et al. 1998a). Evergreen trees also tend to exhibit stronger suppression of photosynthesis (Yamori et al. 2014; Reich et al. 2015, 2018; Crous et al. 2022) and growth (Reich et al. 2015, 2018) in response to warming than deciduous trees, as do boreal than temperate species growing together at their ecotone (Reich et al. 2015, 2018). Together, this suggests that boreal conifers may experience only modest photosynthetic benefits under warming and EC compared to more substantial increases in photosynthesis in deciduous species. Furthermore, some studies report contrasting responses within these functional groups: EC effects are inconsistent across species, and spruces have a stronger negative response to warming (Wang et al. 1996 at high leaf temperatures; Way and Sage 2008a, 2008b) than do larches (Dusenge et al. 2020; Murphy and Way 2021) and pines (Kurepin et al. 2018). Whether these responses are typical of evergreen and deciduous trees remains unknown. Thus, a mechanistic understanding of the photosynthetic responses to climate change across several boreal tree plant functional groups is necessary if we are to accurately predict CO_2_ exchange between these forests and the atmosphere in a warmer, CO_2_-enriched future (Rogers et al. 2022).

We therefore characterized photosynthetic acclimation of five widespread North American boreal tree species, across three plant functional groups, to EC and warming. Over 70% of Canada's forest cover is represented by the genera investigated in this work, and the five species [white spruce (*Picea glauca*), black spruce (*Picea mariana*), Jack pine (*Pinus banksiana*), tamarack (*Larix laricina*), and paper birch (*Betula papyrifera*)] are widely distributed across Canada and the northern United States (Burns and Honkala 1990; Farrar 1995). White spruce, black spruce, and Jack pine are evergreen needle-leaved conifers, whereas paper birch is a deciduous broad-leaf angiosperm. Tamarack is a deciduous conifer and represents a functional overlap between the needle-leaved species and paper birch. We focused on describing the temperature responses of *V*_cmax_ and *J*_max_ in these species, as these traits are critical for ESMs, but are rarely measured. We had the following predictions:

- (P1) Both *V*_cmax_ and *J*_max_ will be reduced under EC, but *A*_growth_ and the *J*_max_/*V*_cmax_ ratio will still be highest in EC-grown plants. Additionally, evergreen species will respond more weakly to EC than will deciduous species, regardless of whether they are needle-leaved or broad-leaved.
- (P2) *A*_net_ will increase under warming conditions in the deciduous species, but *A*_net_ and photosynthetic capacity will decrease in response to warming in the evergreen species.
- (P3) The *T*_optA_ will increase under warming, and these changes in *T*_optA_ will be linked with adjustments to the basal rates, thermal optima, and activation energies of *V*_cmax_ and *J*_max_.
- (P4) *A*_net_ and photosynthetic capacity will respond in an additive manner to combined EC and warming treatments.

## Results

### Experimental treatments

Air temperatures and [CO_2_] were similar in 2019 and 2021, and the treatments were well maintained throughout the growth periods. In 2019, mean daily temperatures over the growing season, averaged across CO_2_ treatments, ranged 11.9–23.9 °C, 13.4–28.0 °C, and 14.5–32.2 °C in the 0T, 4T, and 8T treatments, respectively, as temperatures shifted from spring to mid-summer and declined into early autumn conditions. Similarly, mean daily temperatures for 2021 ranged 12.1–23.5 °C, 15.9–27.6 °C, and 19.8–31.2 °C in the 0T, 4T, and 8T treatments, respectively. Mean [CO_2_] was 399 ± 52 ppm in AC conditions and 731 ± 48 ppm in EC conditions.

### Net photosynthesis

**Figure 1.**
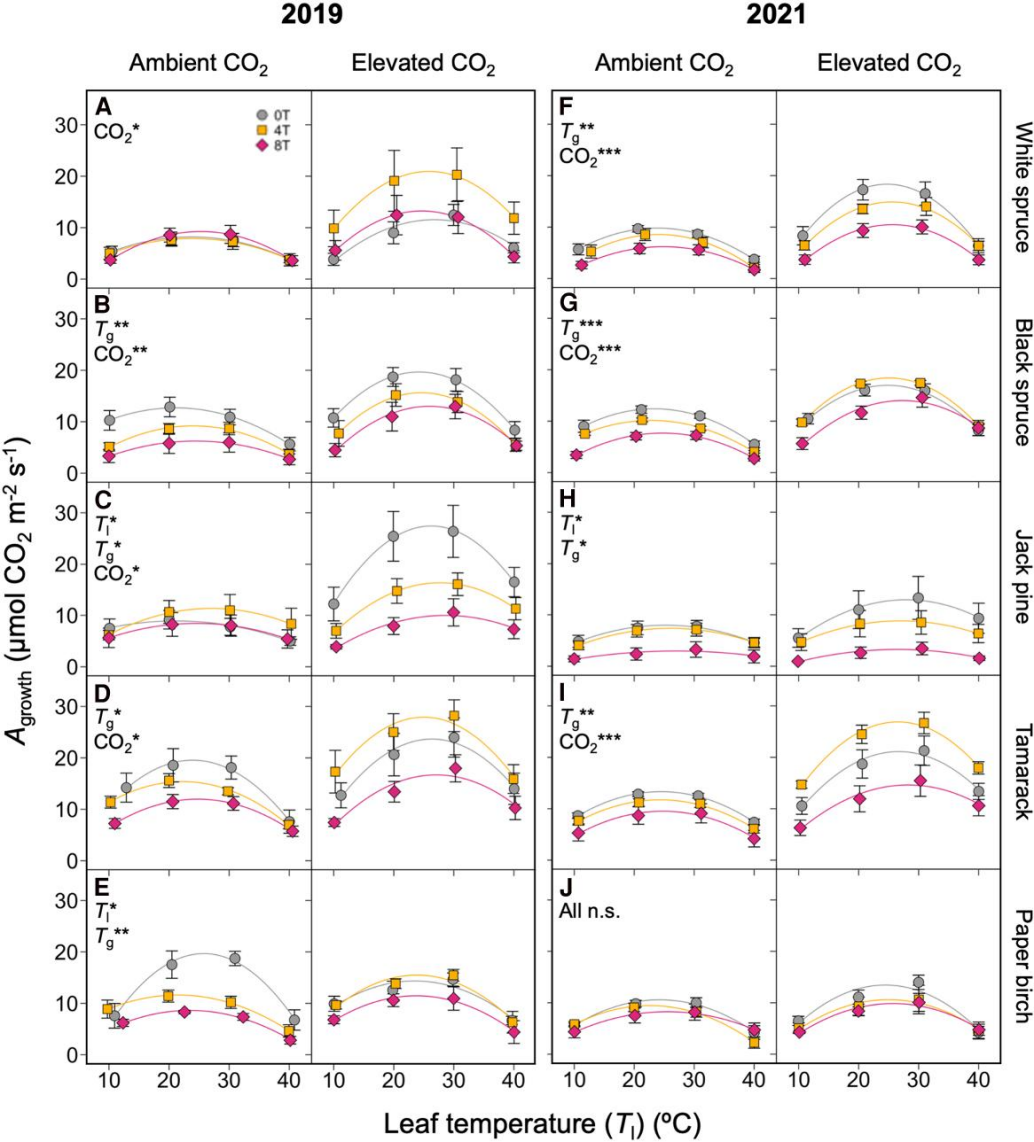
Short-term response to leaf temperature of light-saturated net CO_2_ assimilation rate. Data measured at growth [CO_2_] (*A*_growth_) in 5 boreal tree species grown under either ambient or elevated [CO_2_], and either ambient temperatures (0T, grey circles), ambient +4 °C (4T, yellow squares), or ambient +8 °C (8T, red diamonds), in 2019 (**A**, **B**, **C**, **D** and **E**) and 2021 (**F**, **G**, **H**, **I** and **J**). Repeated measures ANOVAs were performed for each year-species combination: main effects were leaf temperature (*T*_l_), growth temperature (*T*_g_), and CO_2_ environment, with individual tree-*T*_l_ relationships as random effects. Means ± SE, *n* = 3–7 (except in *d*, where *n* = 2–6). *P < 0.05, **P < 0.01, and ***P < 0.001.

Net CO_2_ assimilation measured at growth [CO_2_] (*A*_growth_) generally increased with CO_2_ enrichment, although this was not statistically significant in paper birch or 2021 Jack pine (Fig. 1; Tables 1 and 2). When averaged across species, temperature treatments, and measurement years, and accounting for leaf temperature, *A*_growth_ was 52% higher (3.95 ± 0.51 *µ*mol CO_2_ m^−2^ s^−1^) in EC compared to AC plants. *A*_growth_ was significantly affected by warming in all but two cases (2019 white spruce and 2021 paper birch) (Fig. 1; Tables 1 and 2). Although *A*_growth_ sometimes increased from 0T to 4T, it was always lowest in 8T plants, with 8T plants having 36% lower (4.03 ± 0.63 *µ*mol CO_2_ m^−2^ s^−1^ lower) *A*_growth_ compared to 0T plants (when averaged across species, measurement years, and CO_2_ treatments, and accounting for leaf temperature).

**Table 1.** Summary of repeated-measures ANOVA results for gas exchange and photosynthetic capacity in seedlings grown in 2019

Parameters	White spruce		Black spruce		Jack pine		Tamarack		Paper birch	
Effects	*F*-value	*P*-value	*F*-value	*P*-value	*F*-value	*P*-value	*F*-value	*P*-value	*F*-value	*P*-value
*A*_growth_										
*T*_l_	0.06	0.81	3.02	*0*.*09*	4.48	**0.04**	0.03	0.86	6.10	**0.02**
*T*_g_	1.49	0.25	5.96	**0.009**	4.77	**0.02**	4.94	**0.02**	5.95	**0.008**
CO_2_	5.27	**0.03**	9.82	**0.005**	6.27	**0.02**	6.88	**0.01**	1.15	0.29
*T*_g_ × CO_2_					2.75	*0*.*09*				
*g*_s_										
*T*_l_	5.21	**0.03**	4.98	**0.03**	1.58	0.21	20.4	**<0.0001**	16.8	**0.0001**
*T*_g_	0.46	0.64	1.45	0.26	0.66	0.53	1.97	0.16	3.19	*0*.*06*
CO_2_	0.13	0.72	1.26	0.27	0.95	0.34	0.01	0.94	2.00	0.17
*C*_i_/*C*_a_										
*T*_l_	14.9	**0.0003**	0.03	0.85	38.5	**<0.0001**	29.4	**<0.0001**	10.6	**0.002**
*T*_g_	1.83	0.19	1.38	0.27	4.25	**0.03**	2.31	0.12	6.12	**0.008**
CO_2_	0.17	0.69	0.14	0.71	8.44	**0.009**	2.26	0.15	34.6	**<0.0001**
*T*_l_ × *T*_g_					3.61	**0.03**	3.32	**0.04**	20.0	**<0.0001**
*T*_l_ × CO_2_					16.9	**0.0001**	18.6	**<0.0001**	1.73	0.19
*T*_g_ × CO_2_	4.05	**0.04**			1.25	0.31	0.56	0.58	17.2	**<0.0001**
*T*_l_ × *T*_g_× CO_2_					1.35	0.27	0.96	0.39	12.9	**<0.0001**
*V*_cmax_										
*T*_l_	180	**<0.0001**	182	**<0.0001**	376	**<0.0001**	230	**<0.0001**	73.9	**<0.0001**
*T*_g_	0.96	0.40	4.64	**0.02**	3.34	*0*.*06*	1.80	0.19	9.24	**0.001**
CO_2_	0.22	0.65	0.63	0.44	0.04	0.84	0.89	0.36	31.7	**<0.0001**
*T*_l_ × *T*_g_					8.35	**0.0006**			3.58	**0.03**
*T*_l_ × CO_2_					0.03	0.85			11.7	**0.001**
*T*_g_ × CO_2_					1.84	0.19			10.4	**0.0007**
*T*_l_ × *T*_g_ × CO_2_					5.52	**0.006**			4.51	**0.01**
*J*_max_										
*T*_l_	9.13	**0.004**	5.43	**0.02**	85.1	**<0.0001**	55.7	**<0.0001**	3.22	*0*.*08*
*T*_g_	0.58	0.57	3.17	*0*.*06*	3.52	**0.048**	2.68	*0*.*09*	6.69	**0.005**
CO_2_	0.03	0.87	0.35	0.56	0.92	0.35	0.03	0.87	4.76	**0.04**

Leaf temperature (*T*_l_), growth temperature (*T*_g_), and growth [CO_2_] were fixed effects, and individual trees were random effects. Empty cells represent fixed effects that did not contribute to model fit (estimated by AICc; see Methods). Traits analysed were rates of light-saturated net CO_2_ assimilation measured at growth [CO_2_] (*A*_growth_, µmol CO_2_ m^−2^ s^−1^), stomatal conductance (g_s_, mol H_2_O m^−2^ s^−1^), the ratio of intercellular [CO_2_] to air [CO_2_] (*C_i_*/*C_a_*), the maximum rate of Rubisco carboxylation (V_cmax_, µmol CO_2_ m^−2^ s^−1^), and the maximum rate of electron transport (J_max_, µmol CO_2_ m^−2^ s^−1^). Numbers in bold and italics represent *P* < 0.05 and 0.05 < *P* < 0.1, respectively.

**Table 2.** Summary of repeated-measures ANOVA results for gas exchange and photosynthetic capacity in seedlings grown in 2021

Parameters	White spruce		Black spruce		Jack pine		Tamarack		Paper birch	
Effects	*F*-value	*P*-value	*F*-value	*P*-value	*F*-value	*P*-value	*F*-value	*P*-value	*F*-value	*P*-value
*A*_growth_										
*T*_l_	1.79	0.18	1.16	0.28	5.84	**0.02**	2.65	0.11	0.86	0.36
*T*_g_	8.06	**0.002**	10.4	**0.0005**	3.53	**0.04**	6.68	**0.005**	1.28	0.29
CO_2_	25.4	**<0.0001**	49.9	**<0.0001**	1.32	0.26	32.3	**<0.0001**	1.56	0.22
*T*_g_ × CO_2_							3.32	*0*.*054*		
*g*_s_										
*T*_l_	1.46	0.23	0.0009	0.98	8.60	**0.004**	5.59	**0.02**	1.08	0.30
*T*_g_	2.58	*0*.*09*	2.03	0.15	2.54	*0*.*097*	1.87	0.18	0.30	0.74
CO_2_	0.51	0.48	1.19	0.29	0.86	0.36	0.39	0.54	14.6	**0.0007**
*T*_g_ × CO_2_									2.92	*0*.*07*
*C*_i_/*C*_a_										
*T*_l_	30.2	**<0.0001**	2.76	0.10	3.67	*0*.*06*	34.5	**<0.0001**	6.78	**0.01**
*T*_g_	4.53	**0.02**	4.96	**0.02**	3.78	**0.04**	9.41	**0.001**	1.42	0.26
CO_2_	0.31	0.58	2.47	0.13	3.59	*0*.*07*	0.04	0.84	4.35	**0.047**
*T*_l_ × *T*_g_			5.15	**0.008**			0.41	0.67		
*T*_l_ × CO_2_			31.8	**<0.0001**			21.4	**<0.0001**		
*T*_g_ × CO_2_			0.95	0.40			0.19	0.83	5.83	**0.008**
*T*_l_ × *T*_g_ × CO_2_			3.16	**0.047**			0.67	0.52		
*V*_cmax_										
*T*_l_	117	**<0.0001**	367	**<0.0001**	209	**<0.0001**	558	**<0.0001**	156	**<0.0001**
*T*_g_	9.39	**0.0007**	8.01	**0.002**	3.12	*0*.*06*	5.82	**0.009**	0.57	0.57
CO_2_	0.02	0.89	0.09	0.77	0.90	0.35	1.65	0.21	2.84	0.10
*T*_l_ × *T*_g_			1.72	0.19	11.4	**<0.0001**	7.69	**0.0009**		
*T*_l_ × CO_2_			0.83	0.37	6.92	**0.01**	3.96	*0*.*050*		
*T*_g_ × CO_2_			4.85	**0.02**	0.06	0.94	2.15	0.14		
*T*_l_ × *T*_g_ × CO_2_			5.42	**0.006**	0.89	0.41	3.36	**0.04**		
*J*_max_										
*T*_l_	0.47	0.50	10.8	**0.002**	81.8	**<0.0001**	78.9	**<0.0001**	11.0	**0.001**
*T*_g_	9.75	**0.0005**	14.5	**0.0001**	3.11	*0*.*06*	5.40	**0.01**	2.20	0.13
CO_2_	0.16	0.69	0.22	0.65	0.25	0.62	1.39	0.25	3.92	*0*.*06*
*T*_g_ × CO_2_			4.16	**0.03**						

Leaf temperature (*T*_l_), growth temperature (*T*_g_), and growth [CO_2_] were fixed effects, and individual trees were random effects. Empty cells represent fixed effects that did not contribute to model fit (estimated by AICc; see Methods). Traits analysed were rates of light-saturated net CO_2_ assimilation measured at growth CO_2_ (*A*_growth_, µmol CO_2_ m^−2^ s^−1^), stomatal conductance to water vapour (g_s_, mol H_2_O m^−2^ s^−1^), the ratio of intercellular [CO_2_] to air [CO_2_] (*C_i_/C_a_*), the maximum rate of Rubisco carboxylation (*V*_cmax_, µmol CO_2_ m^−2^ s^−1^), and the maximum rate of electron transport (*J*_max_, µmol CO_2_ m^−2^ s^−1^). Numbers in bold and italics represent *P* < 0.05 and 0.05 < *P* < 0.1, respectively.

**Figure 2.**
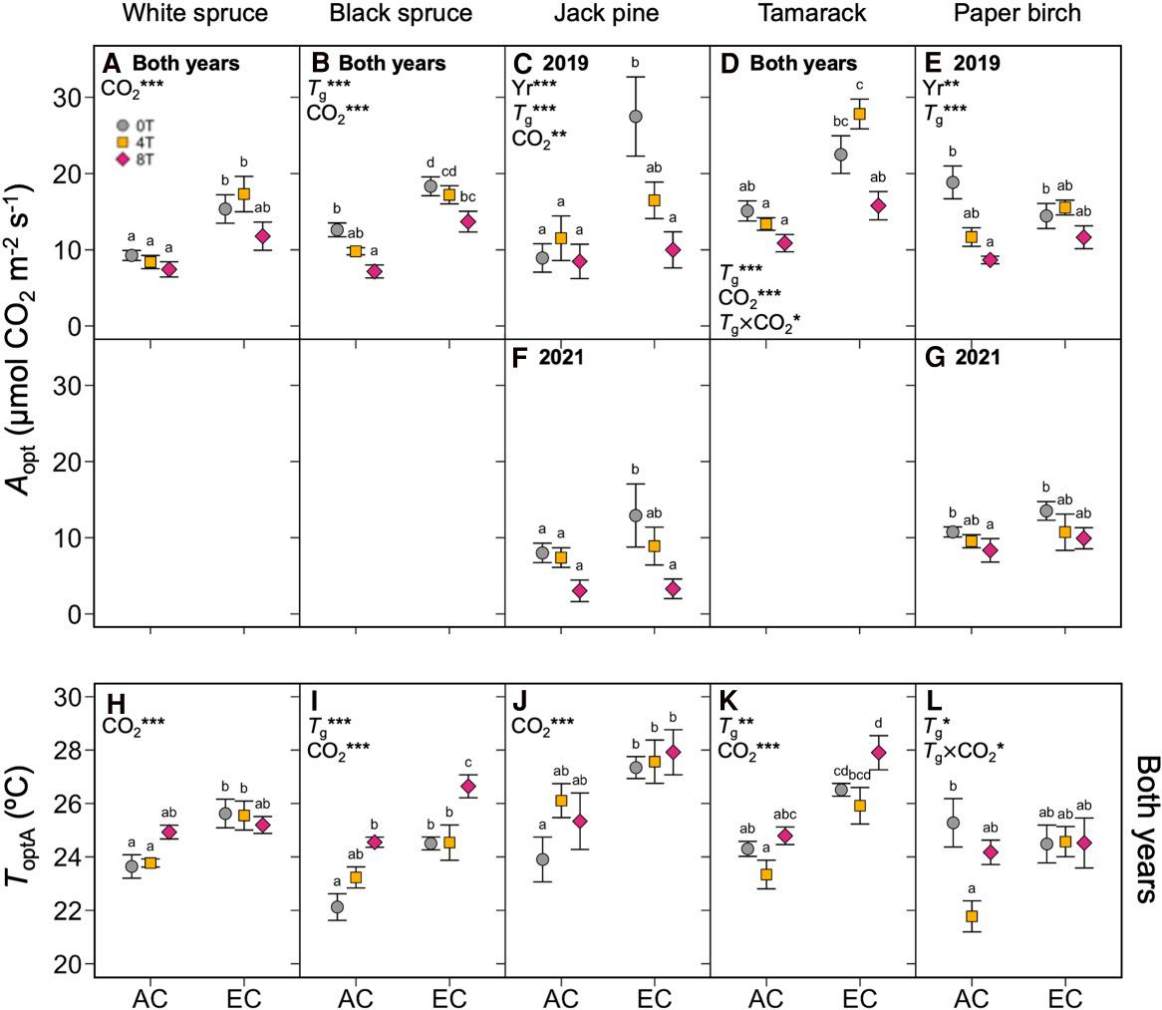
Maximum rates of *A*_growth_ and thermal optima of *A*_growth._ The *A*_growth_ (*A*_opt_, top panels) and thermal optima of *A*_growth_ (*T*_optA_, bottom panels) measured in five boreal tree species grown under either ambient (AC) or elevated CO_2_ (EC), and either ambient temperatures (0T, grey circles), ambient +4 °C (4T, yellow squares), or ambient +8 °C (8T, red diamonds) in 2019 and 2021. Three-way ANOVAs were performed on each parameter for each species, with measurement year (Yr), growth temperature (*T*_g_) and CO_2_ environment as main effects. Measurement year improved model fit for *A_opt_* in Jack pine **C** and **F**) and Paper birch **E** and **G)**. Measurement year did not improve model fit for *A*_opt_ in white spruce **A)**, black spruce **B)**, or tamarack **D)**, nor for *T*_optA_ in all species (**H–L**), so data were combined across years and two-way ANOVAs with only *T*_g_ and CO_2_ as main effects were performed. Letters represent post-hoc Tukey comparisons across the 6 treatments (*P* < 0.05). Means ± SE, n = 3–12. *P < 0.05, **P < 0.01, and ***P < 0.001.

Similarly, maximum rates of *A*_growth_ (*A*_opt_) were typically higher in EC- and cooler-grown seedlings (Fig. 2A–G; Table 3). The only exception to these broad trends was paper birch, for which neither *A*_growth_ nor *A*_opt_ responded to EC conditions. Seedlings also shifted the thermal optimum of *A*_growth_ (*T*_optA_) in response to growth conditions (Fig. 2): *T*_optA_ was 1.84 ± 0.30 °C higher in EC compared to AC plants when averaged within species and warming treatments, and across measurement years. The ANOVA showed that *T*_optA_ also shifted under warming in black spruce, tamarack, and paper birch (Table 3), but there was only a significant increase in *T*_optA_ in black spruce according to the post-hoc test (Fig. 2I), where *T*_optA_ increased by roughly 0.29 °C per 1 °C warming. Significant interactions between growth [CO_2_] and temperature were observed in only tamarack and paper birch: *A*_opt_ decreased with warming in AC tamarack but not the 0T and 4T EC-grown tamarack (Fig. 2D), and *T*_optA_ decreased from 0T to 4T in AC-grown paper birch but was unaffected by warming in EC-grown plants (Fig. 2L).

**Table 3.** Summary of ANOVA results for short-term temperature response parameters and leaf traits

Parameters	White spruce		Black spruce		Jack pine		Tamarack		Paper birch	
Effects	*F*-value	*P*-value	*F*-value	*P*-value	*F*-value	*P*-value	*F*-value	*P*-value	*F*-value	*P*-value
*A*_opt_										
Yr					15.4	**0.0003**			10.9	**0.002**
*T*_g_	2.54	*0*.*09*	11.3	**0.0001**	8.48	**0.0007**	10.4	**0.0002**	9.40	**0.0003**
CO_2_	28.5	**<0.0001**	55.6	**<0.0001**	8.74	**0.005**	36.0	**<0.0001**	2.26	0.14
*T*_g_ × CO_2_	1.19	0.31	0.33	0.72	3.04	*0*.*06*	3.73	**0.03**	1.22	0.30
*T*_optA_										
Yr							2.86	*0*.*097*		
*T*_g_	1.03	0.37	13.1	**<0.0001**	1.14	0.33	6.08	**0.004**	3.50	**0.04**
CO_2_	16.9	**0.0001**	27.6	**<0.0001**	14.6	**0.0004**	45.3	**<0.0001**	2.21	0.14
*T*_g_ × CO_2_	2.83	*0*.*07*	0.78	0.46	0.91	0.41	0.19	0.83	3.49	**0.04**
*V*_cmaxopt_										
Yr	3.80	*0*.*06*			19.1	**0.0001**			3.69	*0*.*06*
*T*_g_	3.72	**0.03**	8.44	**0.0007**	8.20	**0.0009**	4.18	**0.02**	7.50	**0.002**
CO_2_	0.23	0.63	0.27	0.61	1.06	0.31	0.11	0.74	25.6	**<0.0001**
Yr × *T*_g_									5.95	**0.005**
Yr × CO_2_									6.62	**0.01**
*T*_g_ × CO_2_	0.26	0.77	4.11	**0.02**	1.54	0.22	1.65	0.20	10.1	**0.0003**
Yr × *T*_g_ × CO_2_									7.31	**0.002**
*J*_maxopt_										
Yr					14.1	**0.0005**	11.7	**0.001**	10.1	**0.003**
*T*_g_	2.50	*0*.*09*	8.91	**0.0005**	6.92	**0.002**	6.46	**0.003**	9.88	**0.0003**
CO_2_	0.0001	0.99	0.41	0.53	0.15	0.70	0.21	0.65	16.1	**0.0002**
*T*_g_ × CO_2_	0.30	0.74	1.47	0.24	2.05	0.14	2.14	0.13	2.40	0.10
*T*_optV_										
Yr	23.4	**<0.0001**			12.6	**0.0009**	4.23	**0.045**		
*T*_g_	3.07	*0*.*06*	0.64	0.53	2.91	*0*.*06*	0.10	0.90	0.37	0.69
CO_2_	0.005	0.94	0.36	0.55	0.90	0.35	0.56	0.46	0.69	0.41
*T*_g_ × CO_2_	3.18	*0*.*05*	3.58	**0.04**	0.24	0.79	0.54	0.59	0.07	0.93
*T*_optJ_										
Yr	28.7	**<0.0001**								
*T*_g_	1.89	0.16	2.89	*0*.*07*	1.56	0.22	2.53	*0*.*09*	0.60	0.55
CO_2_	0.92	0.34	0.003	0.96	0.34	0.56	1.38	0.25	0.10	0.75
*T*_g_ × CO_2_	7.84	**0.001**	0.96	0.39	0.10	0.90	0.46	0.64	0.46	0.63
*E*_aV_										
Yr					9.74	**0.003**				
*T*_g_	3.43	**0.04**	2.98	*0*.*06*	1.22	0.30	4.16	**0.02**	1.80	0.18
CO_2_	0.22	0.64	1.10	0.30	0.12	0.73	0.01	0.92	0.04	0.85
*T*_g_ × CO_2_	1.96	0.15	0.27	0.76	0.32	0.73	0.56	0.57	5.46	**0.007**
*E*_aJ_										
Yr							5.77	**0.02**		
*T*_g_	2.49	*0*.*09*	5.33	**0.008**	0.17	0.85	1.93	0.16	2.44	*0*.*098*
CO_2_	3.26	*0*.*08*	0.26	0.61	0.17	0.68	0.66	0.42	1.93	0.17
*T*_g_ × CO_2_	1.40	0.26	0.88	0.42	0.78	0.47	0.81	0.45	5.45	**0.007**
*J*_max20_/*V*_cmax20_										
Yr	9.94	**0.003**	7.77	**0.008**					1.00	0.32
*T*_g_	0.90	0.41	2.59	*0*.*09*	0.75	0.48	0.14	0.87	6.79	**0.003**
CO_2_	1.68	0.20	2.07	0.16	2.27	0.14	9.77	**0.003**	27.4	**<0.0001**
Yr × *T*_g_									4.46	**0.02**
Yr × CO_2_									6.87	**0.01**
*T*_g_ × CO_2_	0.86	0.43	1.18	0.32	0.04	0.96	0.64	0.53	4.17	**0.02**
Yr × *T*_g_ × CO_2_									3.68	**0.03**
*N*_a_										
Yr	5.20	**0.03**	10.5	**0.002**	35.9	**<0.0001**	17.8	**0.0001**		
*T*_g_	20.5	**<0.0001**	23.9	**<0.0001**	11.0	**0.0001**	2.96	*0*.*06*	0.97	0.39
CO_2_	0.02	0.88	0.03	0.86	0.04	0.85	3.24	*0*.*08*	0.03	0.87
*T*_g_ × CO_2_	0.99	0.38	2.79	*0*.*07*	0.12	0.89	0.22	0.80	2.29	0.11
LMA										
Yr	79.7	**<0.0001**	141	**<0.0001**	146	**<0.0001**	193	**<0.0001**	19.8	**0.0001**
*T*_g_	22.0	**<0.0001**	12.0	**0.0001**	14.8	**<0.0001**	3.93	**0.03**	2.79	*0*.*07*
CO_2_	0.15	0.70	3.79	*0*.*06*	0.45	0.50	7.91	**0.007**	7.28	**0.01**
*T*_g_ × CO_2_	0.16	0.85	4.37	**0.02**	0.25	0.78	3.64	**0.03**	3.44	**0.04**

Main effects were measurement year (Yr), growth temperature (*T*_g_), and growth [CO_2_]. Empty cells represent fixed effects that did not contribute to model fit (estimated by AICc; see Methods). Traits analysed were the maximum rate of *A*_growth_ (*A*_opt_, µmol CO_2_ m^−2^ s^−1^), the thermal optimum of A_growth_ (T_optA_, °C), the maximum rates of *V*_cmax_ (*V*_cmaxopt_, µmol CO_2_ m^−2^ s^−1^), and *J*_max_ (*J*_maxopt_, µmol CO_2_ m^−2^ s^−1^), the thermal optimums of *V*_cmax_ (*T*_optV_, °C) and *J*_max_ (*T*_optJ_, °C), the activation energies of *V*_cmax_ (*E*_aV_, J mol^−1^) and *J*_max_ (E_aJ_, J mol^−1^), *V*_cmax_ and *J*_max_ measured at 20 °C (*V*_cmax20_ and *J*_max20_, µmol CO_2_ m^−2^ s^−1^), the ratio of *J*_max20_ to *V*_cmax20_ (*J*_max20_/*V*_cmax20_), leaf nitrogen per unit area (N_a_, g m^−2^), and leaf mass per unit area (LMA, g m^−2^). Numbers in bold and italics represent *P* < 0.05 and 0.05 < *P* < 0.1, respectively.

Stomatal conductance (*g*_s_) measured at growth [CO_2_] was generally unaffected by the treatments (Supplementary Fig. S1; Tables 1 and 2), except in paper birch, where EC reduced *g*_s_ in 2021 (Supplementary Fig. S1J; Table 2). While *g*_s_ responded significantly to growth temperature in some cases, these changes were relatively small, differing by less than 0.10 mol H_2_O m^−2^ s^−1^ on average within each year-species-treatment combination.

The ratio between intercellular [CO_2_] and ambient [CO_2_] (*C*_i_/*C*_a_) indicates the balance between CO_2_ supply (*g*_s_) and CO_2_ demand (photosynthetic capacity). There was substantial variation among species and measurement years in how *C*_i_/*C*_a_ responded to growth environment (Tables 1 and 2), though absolute changes in *C*_i_/*C*_a_ were small: average *C*_i_/*C*_a_ was 0.72 ± 0.01 across all measurement years, species, treatments, and leaf temperatures (Supplementary Fig. S2). Nevertheless, *C*_i_/*C*_a_ was usually highest in the 8T seedlings.

### Photosynthetic capacity

**Figure 3.**
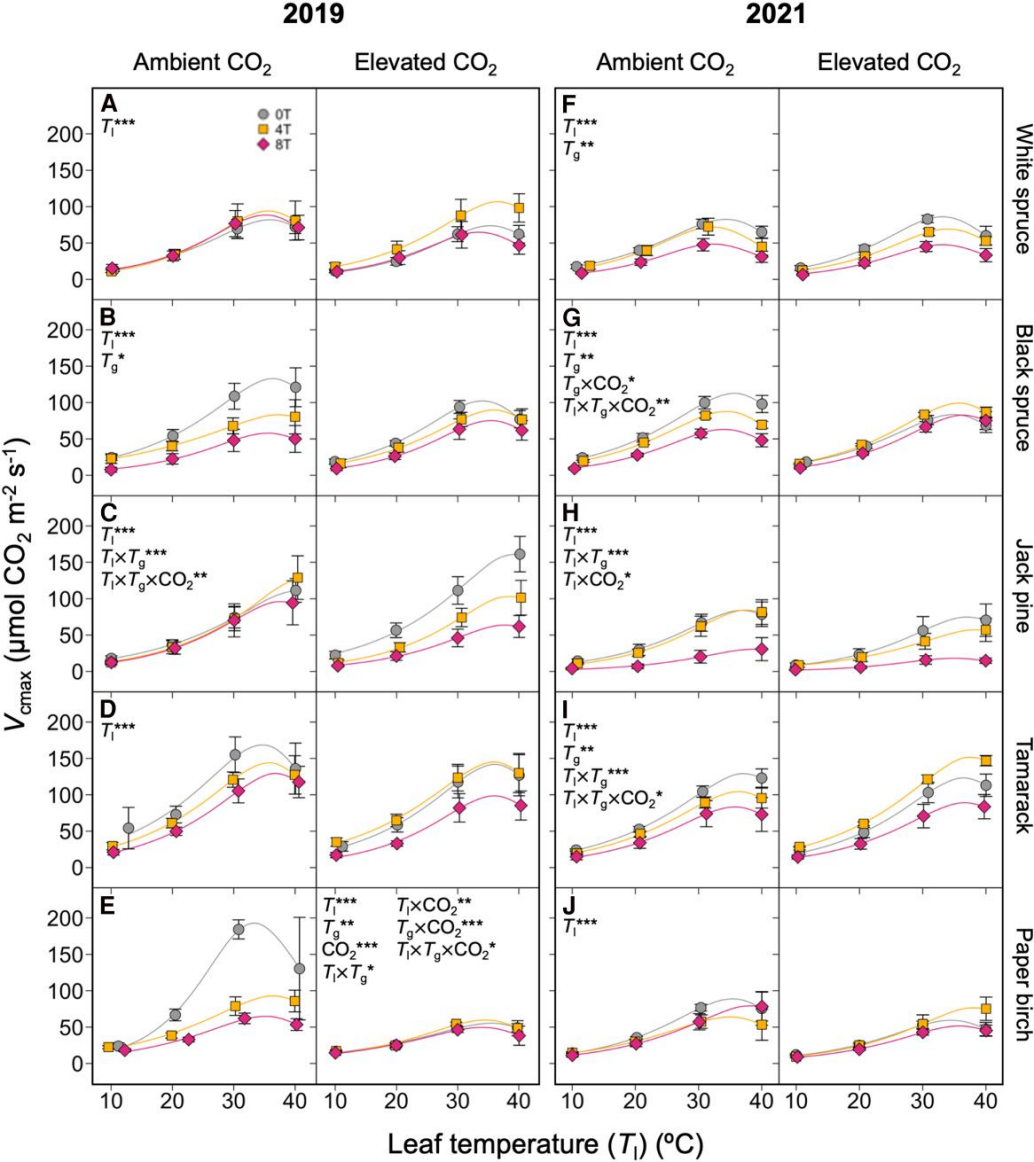
Short-term response to leaf temperature of the maximum rate of Rubisco carboxylation (*V*_cmax_). Data measured in 5 boreal tree species grown under either ambient or elevated [CO_2_], and either ambient temperatures (0T, grey circles), ambient +4 °C (4T, yellow squares), or ambient +8 °C (8T, red diamonds), in 2019 (**A–E**) and 2021 (**F–J**). Repeated measures ANOVAs were performed for each year-species combination: main effects were leaf temperature (*T*_l_), growth temperature (*T*_g_), and CO_2_ environment, with individual tree-*T*_l_ relationships as random effects. Means ± SE, *n* = 3–7 (except in d and e, where *n* = 2–6). *P < 0.05, **P < 0.01, and ***P < 0.001.

The response of maximum rates of Rubisco carboxylation (*V*_cmax_) to leaf temperature, growth temperature, and growth [CO_2_] varied across species and measurement years (Fig. 3; Tables 1 and 2). The *V*_cmax_ generally declined in warm-grown plants, with values typically being lowest in 8T plants. This warming-induced decrease in *V*_cmax_ was stronger at higher leaf temperatures, resulting in significant leaf temperature by growth temperature interaction effects. The response of *V*_cmax_ to warming was sometimes altered by growth [CO_2_]: *V*_cmax_ was reduced under in AC- but not EC-grown 2019 paper birch and 2021 black spruce (Fig. 3E, G), whereas the opposite occurred in 2019 Jack pine (Fig. 3C). Interestingly, *V*_cmax_ was increased under warming in EC-grown but not AC-grown tamarack (Fig. 3D, I), and *V*_cmax_ was not affected by either elevated growth temperature or [CO_2_] in 2019 white spruce and 2021 paper birch (Fig. 3A, J).

**Figure 4.**
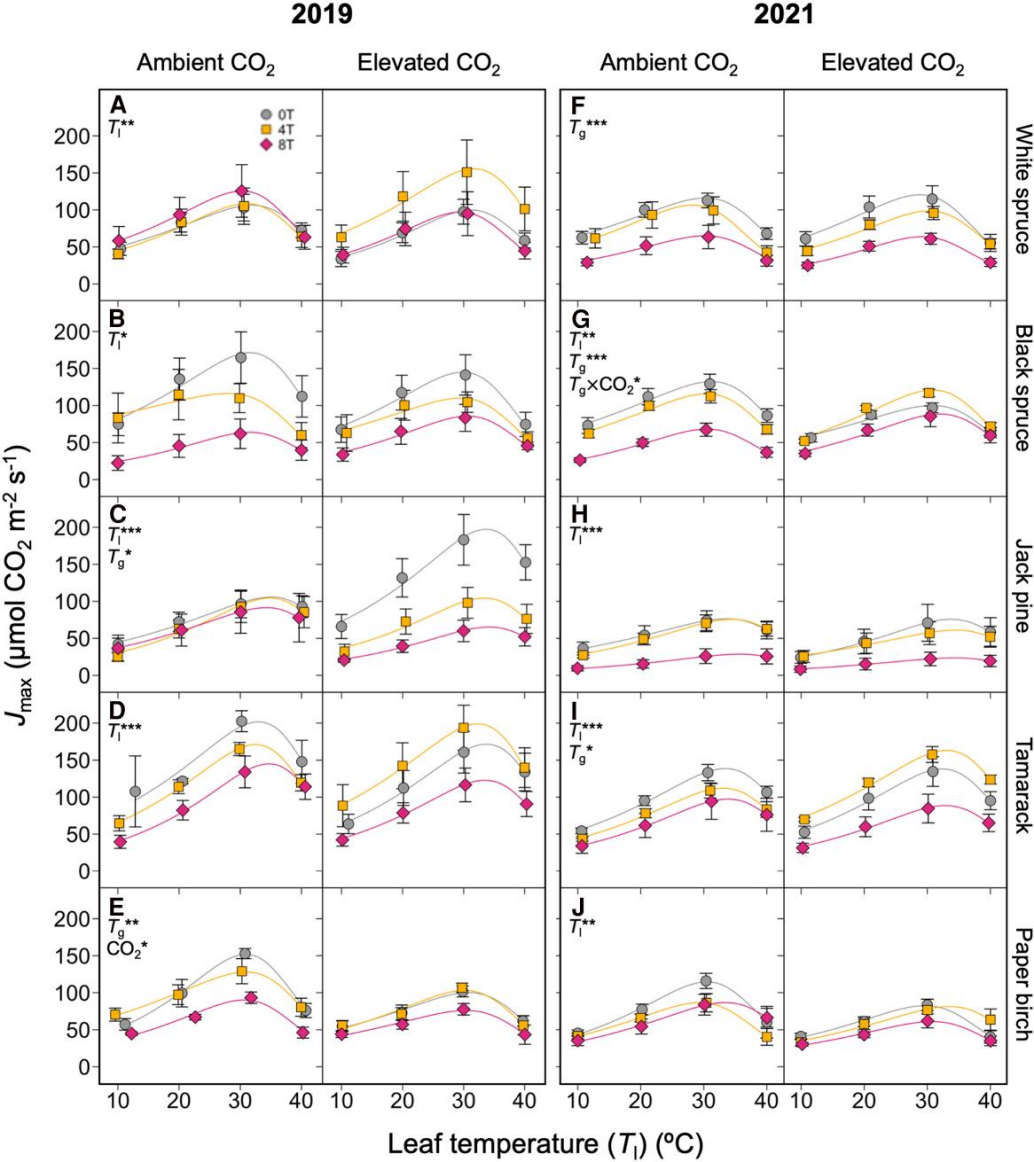
Short-term response to leaf temperature of the maximum rate of electron transport (*J*_max_). Data measured in 5 boreal tree species grown under either ambient or elevated [CO_2_], and either ambient temperatures (0T, grey circles), ambient +4 °C (4T, yellow squares), or ambient +8 °C (8T, red diamonds), in 2019 (**A–E**) and 2021 (**F–J**). Repeated measures ANOVAs were performed for each year-species combination: main effects were leaf temperature (*T*_l_), growth temperature (*T*_g_), and CO_2_ environment, with individual tree-*T*_l_ relationships as random effects. Means ± SE, *n* = 3–7 (except in d and e, where *n* = 2–6). *P < 0.05, **P < 0.01, and ***P < 0.001.

Maximum rates of electron transport (*J*_max_) were less variable than *V*_cmax_ in response to changes in growth environment. As with *V*_cmax_, *J*_max_ generally increased with leaf temperature up to an optimum temperature and decreased with growth temperature (Fig. 4; Tables 1 and 2). The *J*_max_ was only affected by growth [CO_2_] in two species: EC led to a reduction in *J*_max_ in the 2019 paper birch and cancelled the warming-induced reduction of *J*_max_ seen in AC-grown black spruce in 2021 (Fig. 4E, G). In many cases, *J*_max_ was altered by only leaf temperature and not by the growth treatments (e.g. 2019 white spruce, black spruce, and tamarack, and 2021 Jack pine and paper birch: Fig. 4A, B, D, H, and I). The sensitivity analysis using the Rubisco kinetics of *Oryza sativa* and *Solanum tuberosum* showed similar response patterns to the data parameterized with tobacco kinetics (Supplementary Fig. S3), suggesting that the results are not artefacts of the selected Rubisco kinetic parameters.

**Figure 5.**
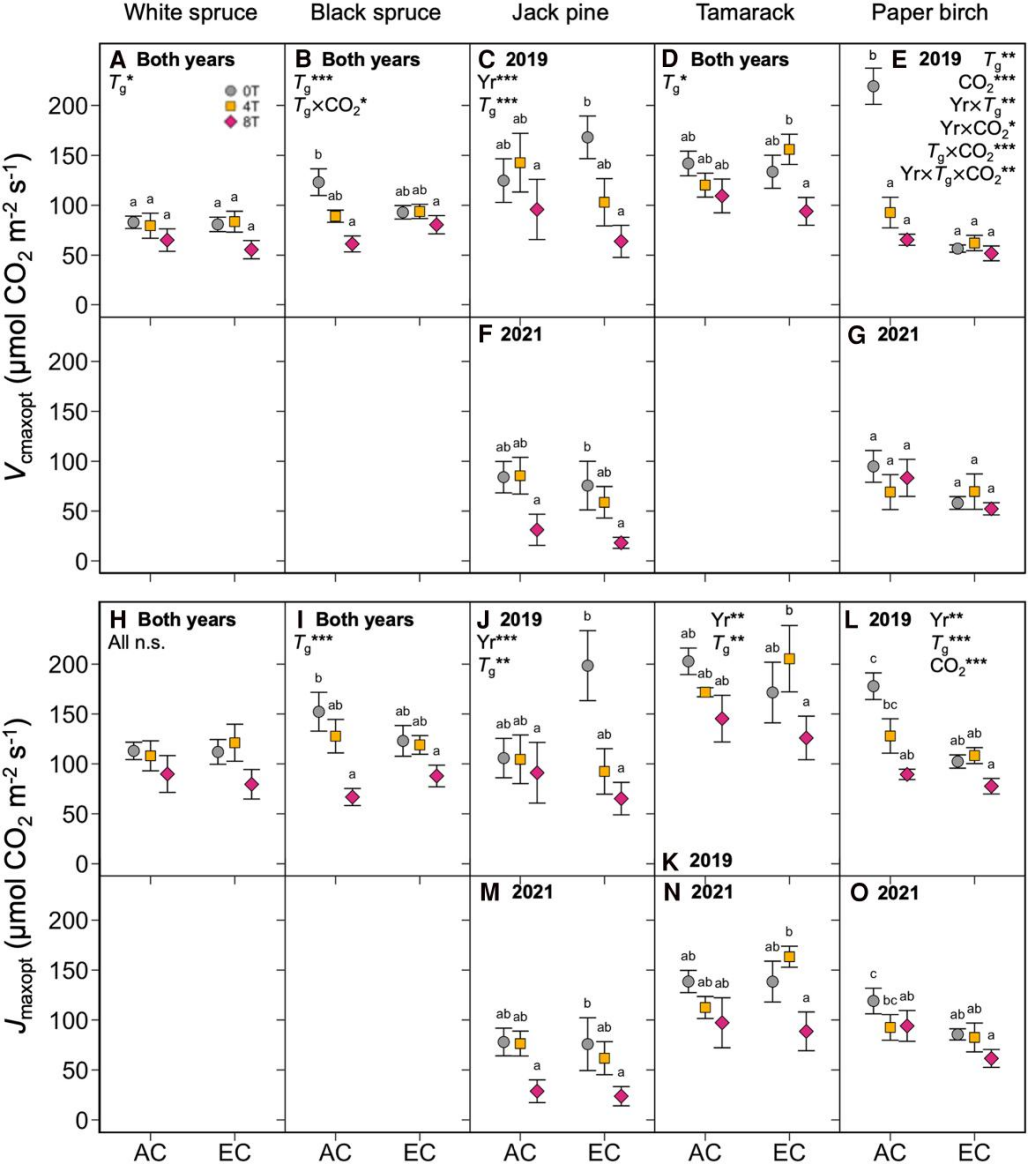
Maximum rates of *V*_cmax_ and *J*_max_. Measurements of the maximum rates of *V*_cmax_ (*V*_cmaxopt_, top panels) and *J*_max_ (*J*_maxopt_, bottom panels) in 5 boreal tree species grown under either ambient (AC) or elevated CO2 (EC), and either ambient temperatures (0T, grey circles), ambient +4 °C (4T, yellow squares), or ambient +8 °C (8T, red diamonds) in 2019 and 2021. Three-way ANOVAs were performed on each parameter for each species, with measurement year (Yr), growth temperature (*T*_g_), and CO_2_ environment as main effects. Measurement year improved model fit for *V_cmaxopt_* and *J_maxopt_* in Jack pine **C**, **F**, **J**, **M**) and Paper birch **E, G, L, O**), and for *J_maxopt_* in Tamarack **K**, **N**). Measurement year did not improve model fit for *V*_cmaxopt_ and/or *J*_maxopt_ in white spruce **A**, **H)**, black spruce **B**, **I)**, or tamarack **D)**, so data were combined across years and two-way ANOVAs with only *T*_g_ and CO_2_ as main effects were performed. Letters represent post-hoc Tukey comparisons across the 6 treatments (*P* < 0.05). Means ± SE, *n* = 3–11 (except in *k*, where *n* = 2–6). *P < 0.05, **P < 0.01, and ***P < 0.001.

**Figure 6.**
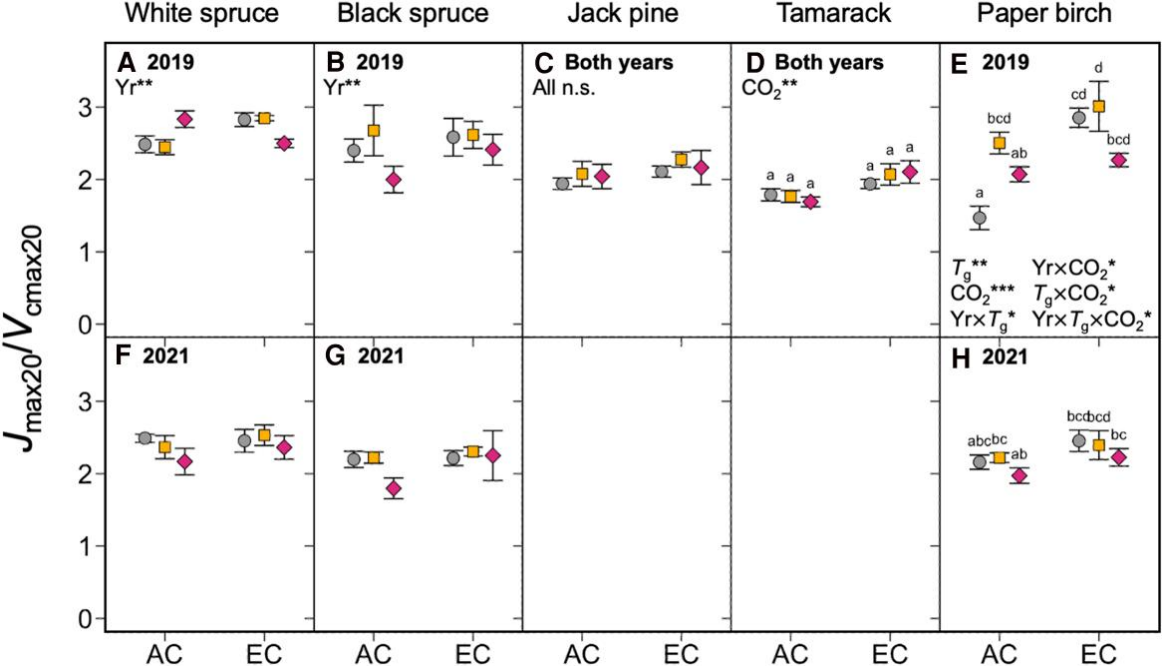
The ratio of *J*_max_ to *V*_cmax_ measured at 20 °C (*J*_max20_/*V*_cmax20_). Data measured in five boreal tree species grown under either ambient (AC) or elevated CO_2_ (EC), and either ambient temperatures (0T, grey circles), ambient +4 °C (4T, yellow squares), or ambient +8 °C (8T, red diamonds) in 2019 and 2021. Three-way ANOVAs were performed for each species, with measurement year (Yr), growth temperature (*T*_g_) and CO_2_ environment as main effects. Measurement year improved model fit for White spruce **A**, **F)**, Black spruce **B**, **G**) and Paper birch **E**, **H**). Measurement year did not improve model fit in Jack pine **C)** and tamarack **D)**, so data were combined across years, and two-way ANOVAs with only *T*_g_ and CO_2_ as main effects were performed. Letters represent post-hoc Tukey comparisons across the 6 treatments (*P* < 0.05). Means ± SE, *n* = 3–12. *P < 0.05, **P < 0.01, and ***P < 0.001.

**Figure 7.**
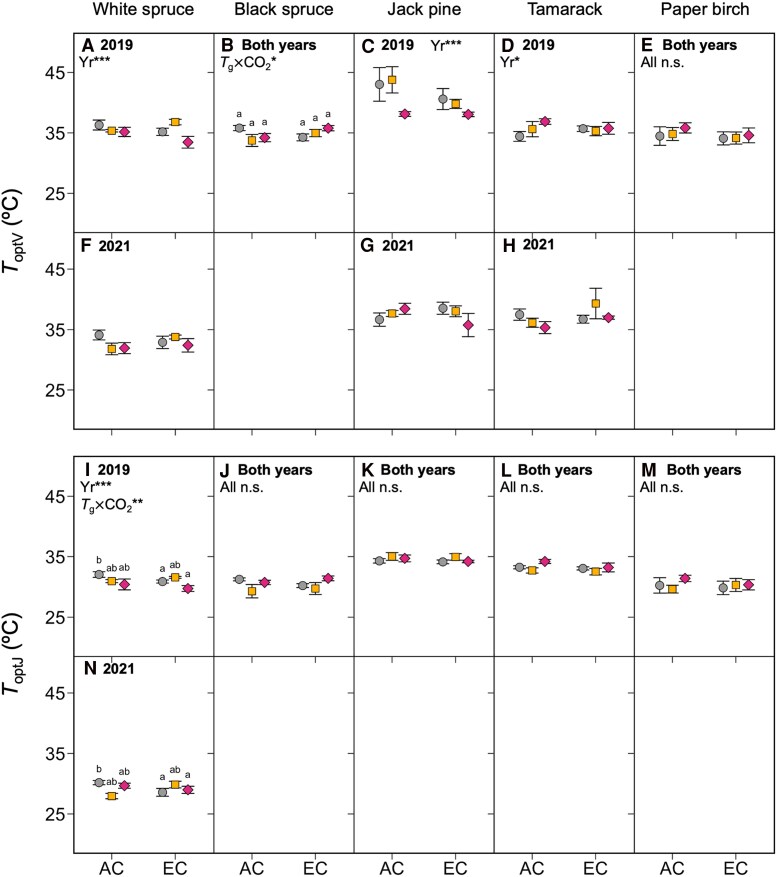
Thermal optima of *V*_cmax_ and *J*_max._ Thermal optima of *V*_cmax_ (*T*_optV_, top panels) and *J*_max_ (*T*_optJ_, bottom panels) in five boreal tree species grown under either ambient (AC) or elevated CO_2_ (EC), and either ambient temperatures (0T, grey circles), ambient +4 °C (4T, yellow squares), or ambient +8 °C (8T, red diamonds) in 2019 and 2021. Three-way ANOVAs were performed on each parameter for each species, with measurement year (Yr), growth temperature (*T*_g_), and CO_2_ environment as main effects. Measurement year improved model fit for *T_optV_* in White spruce **A**, **F**), Jack pine **C**, **G**) and Tamarack **D**, **H**). Measurement year did not improve model fit for *T*_optV_ in black spruce **B)** or paper birch **E)** nor for *T*_optJ_ in all species **J–M)** except white spruce **I, N)**, so data were combined across years and two-way ANOVAs with only *T*_g_ and CO_2_ as main effects were performed. Letters represent post-hoc Tukey comparisons across the 6 treatments (*P* < 0.05). Means ± SE, *n* = 3–11 (except in d, where *n* = 2–6). *P < 0.05, **P < 0.01, ***P < 0.001.

**Figure 8.**
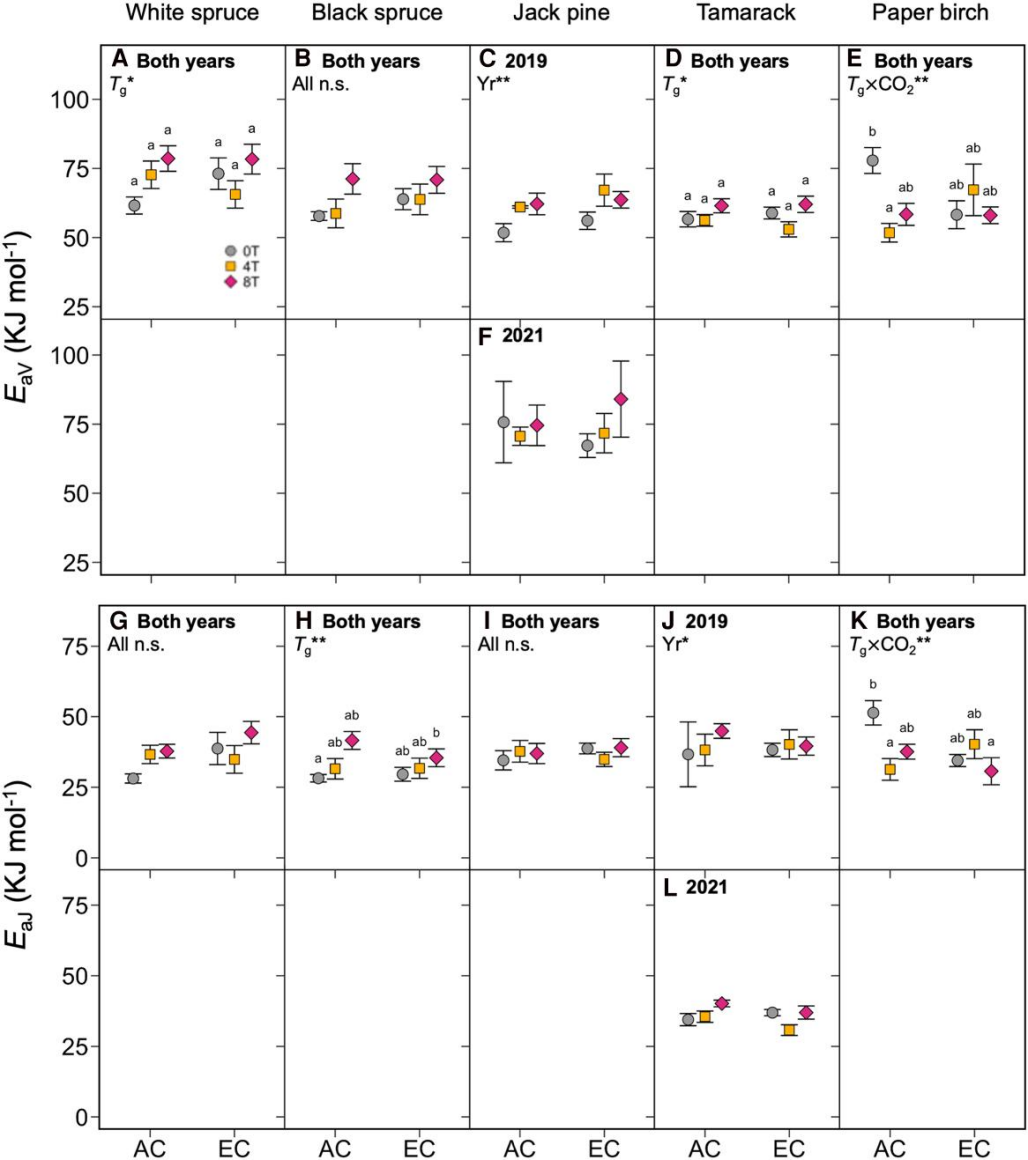
Activation energies of *V*_cmax_ and *J*_max_. Activation energies of *V*_cmax_ (*E*_aV_, top panels) and *J*_max_ (*E*_aJ_, bottom panels) in five boreal tree species grown under either ambient (AC) or elevated CO_2_ (EC), and either ambient temperatures (0T, grey circles), ambient +4 °C (4T, yellow squares), or ambient +8 °C (8T, red diamonds) in 2019 and 2021. Three-way ANOVAs were performed on each parameter for each species, with measurement year (Yr), growth temperature (*T*_g_) and CO_2_ environment as main effects. Measurement year only improved model fit for *E*_aV_ in Jack pine **C, F)** and *E*_aJ_ in tamarack **J, L)**; elsewhere **A**, **B**, **D**, **E**, **G**, **H**, **I**, **K**), data were combined across years and two-way ANOVAs with only *T*_g_ and CO_2_ as main effects were performed. Letters represent post-hoc Tukey comparisons across the 6 treatments (*P* < 0.05). Means ± SE, *n* = 3–11 (except in *j*, where *n* = 2–6). *P < 0.05, **P < 0.01, and ***P < 0.001.

The maximum values of *V*_cmax_ and *J*_max_ (*V*_cmaxopt_ and *J*_maxopt_, respectively) responded to changes in growth temperature and CO_2_ condition in the same way as *V*_cmax_ and *J*_max_: both were generally lower in warm-grown plants and unaffected in plants grown in EC (Fig. 5; Table 3), with few exceptions. *V*_cmax_ and *J*_max_ measured at 20 °C (*V*_cmax20_ and *J*_max20_) followed a similar pattern, resulting in little to no change in the ratio of *J*_max20_/*V*_cmax20_ across the different treatments, except in paper birch, where the ratio of *J*_max20_/*V*_cmax20_ was increased for the 0T trees grown under EC (Fig. 6; Table 3). The thermal optima and activation energies of *V*_cmax_ and *J*_max_ (*T*_optV_, *T*_optJ_, *E*_aV_, and *E*_aJ_, respectively) were also generally unaffected by changes in growth environment (Figs. 7 and 8; Table 3), though in some cases *E*_aV_ or *E*_aJ_ increased with warming (e.g. *E*_aV_ in white spruce, *E*_aJ_ in black spruce, and *E*_aV_ in tamarack: Fig. 8A, D, and H), and the exact values of *E*_aV_ and *E*_aJ_ varied somewhat between the two replicate years.

### Leaf traits

Leaf nitrogen per unit area (*N*_a_), an indication of leaf protein content per unit leaf area, decreased with warming in the evergreen species but was not affected by warming or [CO_2_] in the deciduous species (Supplementary Fig. S4; Table 3). Leaf mass per unit area (LMA), a measure of leaf structural investment, also decreased with warming (Supplementary Fig. S5; Table 3), though this effect was sometimes weakened or absent in EC-grown plants. Leaf mass-based N (%N) decreased with warming in white spruce (*P* < 0.001) and black spruce (*P* < 0.001) and decreased with elevated [CO_2_] in paper birch (*P* < 0.01). This implies that reductions in *N*_a_ under warming were driven by reductions in both %N and LMA in white spruce and black spruce, but were largely driven by lower LMA in Jack pine. All species had higher %N in 2021 compared to 2019 (*P* < 0.001).

## Discussion

We grew five North American boreal tree species under EC and warming to characterise their photosynthetic responses to future climate change. We found partial support for our Predictions of how photosynthetic traits would respond to changes in growth temperature and [CO_2_] across the different species. Net photosynthesis (*A*_growth_ and *A*_opt_) was increased under elevated [CO_2_], which reflected higher CO_2_ supply at the site of photosynthesis, given the generally weak response of photosynthetic capacity (*V*_cmax_ and *J*_max_) and *g*_s_ to EC conditions, in partial support of Prediction 1 (P1). By contrast, *V*_cmax_ and *J*_max_ strongly declined in warm-grown plants across a range of ecologically relevant leaf temperatures with no influence on *g*_s_, leading to overall lower *A*_growth_ and *A*_opt_ in the warmest-grown seedlings. While we predicted such a response to warming for evergreen species in Prediction 2 (P2), this response was also seen in the deciduous species. The higher *C*_i_/*C*_a_ ratios in warmed plants indicate stronger biochemical limitations to photosynthesis than stomatal limitations. Additionally, the thermal optimum of photosynthesis (*T*_optA_) was increased by both EC and warming in some species, although the temperature sensitivities of *V*_cmax_ and *J*_max_ were largely unchanged by the growth treatments, in partial support of Prediction 3 (P3). Interactive effects between growth [CO_2_] and temperature were rare, in line with Prediction 4 (P4), and treatment responses of photosynthetic parameters were generally consistent across species.

### Photosynthetic responses to elevated [CO_2_] effects

Contrary to our hypothesis, *V*_cmax_ and *J*_max_ responded only weakly to the EC treatment. This was unexpected, given the numerous reports of photosynthetic acclimation to EC (Drake et al. 1997; Ainsworth and Long 2005; Ainsworth and Rogers 2007; Crous et al. 2008; Norby et al. 2010; Pastore et al. 2019; Saban et al. 2019), including for four of the same species as in our study (Tjoelker et al. 1998a). The accumulation of leaf sugars in CO_2_-enriched plants is thought to downregulate the transcription of genes that encode photosynthetic proteins and enzymes, leading to inhibition of *V*_cmax_ and *J*_max_ (Moore et al. 1999). This negative feedback helps rebalance sugar production (source) with growth (sink) demand and allows plants to conserve scarce nutrients such as nitrogen (Duarte et al. 2021). In an earlier study of four of the species used in our study, strong sugar accumulation was noted under EC, consistent with the strong acclimation of photosynthesis to EC (Tjoelker et al. 1998a). Accordingly, strong sink demand and/or ample nutrient supply in the seedlings studied here may have suppressed these feedbacks, resulting in minimal photosynthetic acclimation to EC. Indeed, nutrient manipulation studies on EC-grown plants report lower inhibition of photosynthetic capacity in fertilised versus non-fertilized treatments (Ainsworth et al. 2003). Similarly, free air CO_2_ enrichment (FACE) studies on trees and crops growing in nutrient-poor soils report lower stimulation of photosynthesis with long-term EC than is seen in FACE and chamber-based studies with well-fertilized plants (Long et al. 2006; Norby et al. 2010). Since the plants used in our experiment were well-fertilised seedlings, high sink demand (due to fast seedling growth rates) and nutrient supply may have prevented acclimation (Körner 2006). Notably, leaf *N*_a_ was unaffected by EC in most species, implying that photosynthetic enzyme concentrations were similar between AC- and EC-grown plants. These results add to a growing number of studies (Wang et al. 1996; Klein et al. 2016; Kurepin et al. 2018; Dusenge et al. 2020; Murphy and Way 2021) that suggest boreal conifers exhibit weak photosynthetic acclimation to EC when nutrient availability is high, though this is not necessarily seen in the field (but see Lamba et al. 2018).

While photosynthetic capacity did not acclimate to EC, *A*_growth_ was generally higher in EC- compared to AC-grown plants. Moreover, we observed only minimal stomatal closure in response to EC, which may have allowed for greater stimulation of *A*_growth_. The combined stomatal-photosynthesis model predicts that plants adjust *g*_s_ to maintain a near-optimal *C*_i_/*C*_a_ because this balances photosynthetic CO_2_ uptake with transpirational water costs (Medlyn et al. 2011). Thus, plants grown under EC should reduce *g*_s_ to conserve water (Ainsworth and Rogers 2007), and many plants have lower stomatal density when grown under EC conditions (Stevens et al. 2021). Here, although *C*_i_/*C*_a_ was unaffected by growth treatments across species and measurement years, *g*_s_ was only reduced under EC in the 2021 paper birch.

### Photosynthetic responses to warming

Photosynthesis (both *A*_growth_ and *A*_opt_) was strongly reduced under warming in all species due to reductions in photosynthetic capacity. This contrasts with the common expectation that photosynthesis in plants from cool regions, such as boreal forests, will benefit from long-term warming (Tjoelker et al. 1998b; Stinziano and Way 2014; Way and Oren 2010). Kattge and Knorr (2007) surveyed the effects of growth temperature on *V*_cmax_ and *J*_max_ and found no adjustments to basal rates or activation energies with long-term warming, resulting in higher modelled rates of photosynthesis in warm-grown compared to cool-grown plants. Similarly, Kumarathunge et al. (2019) showed that basal rates of *V*_cmax_ were largely unaffected by growth temperature in field-grown trees, though there was evidence for a warming-induced decline in *J*_max_ in mature plants. However, basal rates of *V*_cmax_ and *J*_max_ can change when plants are exposed to warmer growth temperatures (Way and Oren 2010; Way and Yamori 2014; Kumarathunge et al. 2019; Crous et al. 2022), as summarized in Way and Yamori (2014), who found that 54% of the studies they evaluated (mostly from glasshouses and growth chambers) showed a decrease in basal *V*_cmax_ with warming and 41% showed an increase. Studies on boreal trees also yield conflicting results. Basal rates of *V*_cmax_ and *J*_max_ in black spruce declined in response to warming (Dusenge et al. 2020), but not in tamarack (Murphy and Way 2021) or in several *Pinus* species (Kurepin et al. 2018; Stefanski et al. 2020; Bermudez et al. 2021). The observed decrease in photosynthetic capacity in our study is in line with the least-cost optimality framework, which suggest that under warmer growth conditions, the leaf-level demand to build and maintain photosynthetic protein is reduced as warm-grown plants can optimally fix an equal amount of carbon with relatively less protein than cool-grown plants (Smith and Keenan 2020 and Wang et al. 2020). However, it is likely that the acclimation of photosynthetic capacity overcompensated to override any benefit to the carbon gain, resulting in observed reductions in photosynthesis in warm-grown plants. Nevertheless, more studies are still needed to reconcile observations with the least-cost optimality prediction (Dusenge et al. 2024).

Why might photosynthetic capacity decrease in warm-grown plants? In this experiment, *V*_cmax_ and *J*_max_, including rates at the thermal optimum (*V*_cmaxopt_ and *J*_maxopt_), declined under warming. This reduction in photosynthetic capacity across leaf temperatures could be caused by lower concentrations of photosynthetic enzymes (Yamori et al. 2014; Smith and Keenan 2020; Wang et al. 2020), a response termed qualitative acclimation (Way and Yamori 2014). Enzyme activity increases with rising leaf temperature, meaning lower enzyme concentrations are needed to achieve the same rates of carboxylation and RuBP regeneration in warmed plants compared to control plants at their growth temperature (Way and Yamori 2014; Wang et al. 2020). Indeed, there were lower leaf *N*_a_ and %N in warm-grown white spruce and black spruce, which is often linked with lower concentrations of Rubisco (Yamori et al. 2005). The weak responses of *T*_optV_, *T*_optJ_, *E*_aV_, *E*_aJ_, and *J*_max20_/*V*_cmax20_ to warming also imply that acclimation was primarily quantitative and not qualitative (sensu Way and Yamori 2014), as changes in these parameters could signify more complex adjustments to the electron transport chain and Calvin-Benson cycle (*e*.*g*. heat stable isoforms of Rubisco). While seasonal warming across the year often leads to an increase in *T*_optV_, *T*_optJ_, and *E*_aV_ in field-grown trees (Kumarathunge et al. 2019), our data indicate that these parameters are not always sensitive to warming. Shifts in *T*_optV_ and *E*_aV_ in warm-grown plants can be attributed to expression of thermally stable isoforms of Rubisco (Yamori et al. 2006) and Rubisco activase (Scafaro et al. 2016). Similarly, shifts in *T*_optJ_ and *E*_aJ_ may be caused by higher saturated fatty acid content in thylakoid membranes, which increases rigidity at high temperatures (Zhu et al. 2018), and more frequent cyclic electron transport (Bukhov et al. 1999), which helps re-establish proton gradients across leaky membranes. Although *T*_optV_, *T*_optJ_, *E*_aV_, *E*_aJ,_ and the ratio of *J*_max_/*V*_cmax_ were unaffected by growth treatment in this experiment, it remains unclear to what extent these strategies are employed by boreal trees. More generally, it is also unknown for how long these processes persist under long-term warming, or whether they are affected by variable daily temperatures, since many of these mechanisms have been studied in only heat-shocked plants.

The reductions in *A*_growth_ seen under the warming treatment were not related to adjustments of *g*_s_. Stomatal conductance does not necessarily respond to temperature per se (e.g. von Caemmerer and Evans 2015), though a temperature effect is seen in some studies (Freeden and Sage 1999; Urban et al. 2017; Kostaki et al. 2020), but more often shifts in response to the leaf-to-air vapour pressure deficit (VPD) (Grossiord et al. 2020; López et al. 2021), which increases with rising temperature. Indeed, the mean daily VPD in 2021 was 1.56 kPa in the 8T treatments, and only 0.72 kPa in the controls. A high VPD stimulates transpiration, such that plants typically reduce *g*_s_ to conserve water (Grossiord et al. 2020). This reduction in *g*_s_ limits CO_2_ diffusion into the leaf, which could suppress *A*_growth_ in warm-grown plants. Although stomatal limitations to photosynthesis directly related to long-term warming are rare (Kumarathunge et al. 2019), *g*_s_ declines with higher VPD, regardless of soil moisture status (Reich et al. 2018), and acclimates to VPD, with high VPD-grown plants exhibiting lower *g*_s_ than their low VPD-grown counterparts (López et al. 2021). Here, *g*_s_ was largely unresponsive to growth temperature/growth VPD, which contrasts with previous work in boreal trees (Way and Sage 2008a; Kroner and Way 2016; Lamba et al. 2018; Reich et al. 2018; Dusenge et al. 2020, 2021). It is possible that *g*_s_ remained high in the warmer-grown plants to enable leaf cooling via evapotranspiration, as seen in heat-stressed *Eucalyptus parramattensis* (Drake et al. 2018). Additionally, boreal field-warming studies find that soil moisture may alter the effect of warming on *g*_s_, where *g*_s_ increases under warming in moist soils but decreases under warming in dry soils (Reich et al. 2018), resulting in a more conservative water-carbon trade-off under warmed conditions (Stefanski et al. 2023). Thus, stomatal responses may be important for photosynthetic responses to temperature in field-grown boreal trees, where VPD and soil moisture effects will be common.

### Responses of the photosynthetic thermal optimum to warming and elevated [CO_2_]

Increases in *T*_optA_ are one of the most common responses of photosynthesis to a warmer growth environment (Way and Yamori 2014). Yet this was not consistently seen in our results. Although *T*_optA_ was generally highest in 8T plants, adjustments to *T*_optA_ in the warming treatment were only significant in black spruce, tamarack, and paper birch, and *T*_optA_ was largely unaffected by moderate warming in the latter two species. For the 4T trees, the lack of response may be linked to the inability to detect small shifts in *T*_optA_ (predicted to be only 1–1.3 °C for +4 °C warming) given our sample sizes. However, in paper birch, we found no evidence for a consistent shift in *T*_optA_. When we did see a higher *T*_optA_ in warm-grown seedlings, the shifts (e.g. 0.29 °C per 1 °C warming in black spruce) were in line with values reported in the literature (0.38 °C per 1 °C warming across C3 plants: Yamori et al. 2014; 0.26–0.38 °C per 1 °C warming for field-warmed boreal and temperate trees: Sendall et al. 2015; and Dusenge et al. 2023). Additionally, we found a higher *T*_optA_ in EC- versus AC-grown plants, as expected under low photorespiratory measurement conditions, and similar to the results of Dusenge et al. (2023) in field-grown tamarack and black spruce. How widespread such responses are, and how powerfully they will help plants compensate for rising temperatures in a warming world, are not clear.

The hypothesis that *T*_optA_ would increase with warming due to shifts in the short-term temperature responses of *V*_cmax_ and *J*_max_ was also not supported. Changes to *T*_optV_ and *E*_aV_, and *T*_optJ_ and *E*_aJ_, affect the thermal optima of Rubisco carboxylation- (*A*_c_) and RuBP regeneration-limited (*A*_j_) photosynthesis, respectively (Hikosaka et al. 2006; Kattge and Knorr 2007; Way and Yamori 2014). Because *A*_j_ has a higher thermal optimum than *A*_c_, changes in *J*_max_/*V*_cmax_ shift the temperature range over which *A*_net_ is equal to either *A*_j_ or *A*_c_, thereby altering *T*_optA_. Several reviews identify *E*_aV_ and *J*_max_/*V*_cmax_ as being the most important parameters for estimating the response of photosynthesis to long-term warming (Hikosaka et al. 2006; Kattge and Knorr 2007; Kumarathunge et al. 2019). These reviews report increases in *E*_aV_ and decreases in *J*_max_/*V*_cmax_ in most warm-grown plants, and these patterns are corroborated by evidence in boreal species such as black spruce and tamarack (Dusenge et al. 2020; Murphy and Way 2021). Here, although *T*_optA_ was generally higher in warmer-grown plants, *T*_optV_, *T*_optJ_, *E*_aV_, *E*_aJ,_ and the ratio of *J*_max_/*V*_cmax_ were all unaffected by growth temperature. Why might this be? Stomatal conductance and mitochondrial respiration are also important for determining *T*_optA_. Since there were no strong warming effects on *g*_s_ here, it is more likely that respiratory acclimation was responsible for higher *T*_optA_ (Way and Yamori 2014). The short-term temperature response of mitochondrial respiration is described by a peaked Arrhenius function (Atkin et al. 2005), where warming-induced reductions in both basal rates and activation energies are reported in many species (Atkin and Tjoelker 2003; Zhu et al. 2021), including black spruce, tamarack (Way and Sage 2008a; Dusenge et al. 2020; but not in mature tamarack and black spruce: Dusenge et al. 2021), Scots pine and Norway spruce (Kroner and Way 2016; Kurepin et al. 2018; Lamba et al. 2018).

### The combined response to elevated [CO_2_] and warming

Photosynthetic activity generally did not show an interactive response to growth [CO_2_] and temperature, which supports our hypothesis that combined EC and warming have additive effects on these traits. When comparing *A*_growth_ measured at 20, 24, and 28 °C in 0TAC, 4TEC, and 8TEC plants, respectively, *A*_growth_ was highest in 4TEC plants and similar between 0TAC and 8TEC plants. This suggests that stimulations of photosynthesis under EC (due to weak acclimation of photosynthetic capacity and *g*_s_ to CO_2_ enrichment) were sufficient to compensate for reductions in photosynthesis in response to warming in the 8T plants, and high enough to improve photosynthesis in 4T plants, when compared to 0TAC controls. That said, there were some notable exceptions: in tamarack, *A*_opt_ decreased with warming in AC plants, but increased from 0T to 4T in EC plants. This could result from non-significant stimulations of *V*_cmax_, *J*_max_, and *g*_s_ by moderate warming under EC but not AC conditions, as increases in *g*_s_ under warming have been reported elsewhere in tamarack (Dusenge et al. 2020, 2021). There was also an interaction in the response of LMA to growth temperature and [CO_2_], suggesting that moderate warming combined with high CO_2_ enrichment produced denser leaves. Notably, the vast majority of growth [CO_2_]× warming interactions were found in paper birch, driven by high *V*_cmax_ in 0TAC seedlings grown in 2019 (which was likely the result of tree-by-tree variation). Despite these instances, combined EC and warming generally had independent effects on photosynthetic activity in these boreal tree species.

### Species differences in leaf nitrogen responses

Leaf *N*_a_ generally shifted in concert with photosynthetic capacity, where *N*_a_ was unaffected by growth [CO_2_] but decreased with warming in the evergreen species. In the two deciduous species, however, *N*_a_ was unaffected by either growth temperature or [CO_2_], despite *V*_cmax_ and *J*_max_ being lowest in the warmest-grown plants. Although Rubisco represents a substantial proportion of *N*_a_ (Spreitzer and Salvucci 2002), reductions in *V*_cmax_ without changes to *N*_a_ are possible if leaf nitrogen is re-allocated to non-photosynthetic leaf proteins and enzymes (e.g. heat protective proteins). The highly variable response of *J*_max20_/*V*_cmax20_ to growth conditions in paper birch could indicate that re-allocation of resources, even between carboxylation and RuBP regeneration, is flexible in this species. These results, combined with the hypothesis that nutrient availability may affect photosynthetic acclimation to EC (discussed above), demonstrate the need for more experiments that measure leaf biochemical responses to different combinations of nutrient level, growth [CO_2_], and temperature in boreal trees.

### Implications for boreal forests and earth system models

As predicted, photosynthesis generally showed additive responses to EC and growth temperature in the species measured here. When comparing control (0TAC) plants to those grown under simulated moderate or extreme climate change scenarios, *A*_growth_ was either maintained (8TEC) or increased (4TEC). Thus, moderate climate change—in terms of atmospheric [CO_2_] and temperatures that we may experience in the next 50–100 years—may enhance leaf-level CO_2_ uptake by seedlings in Canadian boreal forests. Importantly, photosynthetic CO_2_ uptake in field-grown boreal trees is influenced by more than just prevailing temperature and [CO_2_]: nutrient availability, precipitation, and growing season length—all of which are expected to change with global warming—are also key considerations for predicting tree growth and survival in the future (Stinziano and Way 2014; Reich et al. 2018; Dusenge et al. 2019). The consistent responses to EC and warming among the species measured here suggest that photosynthetic traits may respond in a broadly similar manner across other boreal tree species at the seedling stage under ample water and nutrient conditions. The general similarities within and among the different plant functional groups studied here provide support for parametrizing boreal trees as one group in terms of photosynthetic responses to climate change in ESMs, which could greatly simplify these models, although more work in natural forests is necessary to confirm that seedling responses are similar to the responses of field-grown trees. While our results suggest limited photosynthetic acclimation responses to elevated CO_2_, strong photosynthetic adjustments to warming indicate that these processes are of high importance for consideration in next-generation ESM model development, as these processes are not included at the process-level in many current model formulations (Rogers et al. 2017).

## Conclusions

Overall, our results offer new knowledge on the photosynthetic responses to combined long-term EC and warming in five widespread North American boreal trees. We demonstrate that photosynthetic capacity in well-watered and well-fertilised seedlings exhibits weak acclimation to long-term EC but is strongly reduced under warming conditions. Generally, photosynthetic processes did not show an interactive response to combined EC and warming, and these responses were similar across species despite their different plant functional groups. These responses were associated with increased leaf-level photosynthesis under moderate climate change and homeostatic photosynthesis under extreme climate change. Together, these findings suggest that models can treat changes in atmospheric [CO_2_] and temperature as having independent effects on photosynthetic CO_2_ uptake across the North American boreal region, though further work is needed to clarify the interactive effects of EC and warming with nutrients, water availability, and tree age, when applying these conclusions to natural forests.

## Materials and methods

### Plant husbandry and experimental design

Three evergreen conifer species (*Picea glauca* [Moench] Voss, white spruce; *Picea mariana* (Mill) BSP., black spruce; and *Pinus banksiana* Lamb., Jack pine), one deciduous conifer species (*Larix laricina* [Du Roi] K. Koch., tamarack), and one deciduous broad-leaf species (*Betula papyrifera* Marsh., paper birch) were grown from seed in two experimental replicates: first in May–October 2019 and then again in May–October 2021 (delayed from 2020 due to the COVID-19 pandemic). Seeds were sourced from between 45 and 46°N in Ontario, Canada (near Algonquin Park and towards the southern ranges of the species) from the Canadian National Tree Seed Centre. These were sown in 11.6 L pots filled with Pro-Mix BX Mycorrhizal growth medium (Premier Tech Home and Garden) and slow-release fertiliser (Slow Release Plant Food, 12-4-8, Miracle Gro, The Scotts Company).

Pots were placed in six rooftop glasshouses at Western University's Biotron Experimental Climate Change Research Centre (43.009°N, 81.274°W). To simulate moderate and extreme climate scenarios (Ranasinghe et al. 2021), each glasshouse was set to a different factorial combination of [CO_2_] and temperature: either ambient (AC, 410 ppm) or elevated (EC, 750 ppm) [CO_2_] and either ambient temperatures (0T), ambient +4 °C (4T), or ambient +8 °C (8T). The elevated CO_2_ of 750 ppm was chosen because it corresponds to atmospheric CO_2_ concentrations projected under an intermediate greenhouse gas emissions scenario. Daily ambient temperatures were set using the five-year (2014–2018) day/night average temperatures for the corresponding day of the year in Algonquin Park, Ontario (45°58′N, 78°48′W) to align with the location of the seed sources. Temperature, [CO_2_], and humidity were controlled by Argus Control Software TITAN version 900 (Surrey, British Columbia, Canada), where pure CO_2_ was added to the air as needed to maintain EC levels, and relative humidity was kept at ∼60%.

Five to ten seeds were sown per pot, and seedlings were thinned to one seedling per pot once they established (around 1–2 months after sowing). Each glasshouse contained 10 seedlings per species in 2019 (total sample size of 300) and 20 seedlings per species in 2021 (total sample size of 600); the experiment originally included 3 extra species in 2019 but the seedlings from those species (*Abies balsamea*, *Populus balsamifera,* and *Populus tremuloides*) did not grow large enough to measure and were excluded in 2021. Pots were watered daily to prevent water stress, and soil moisture was checked weekly using a soil moisture probe (HH2 Moisture Meter, Delta-T Devices, Cambridge, United Kingdom) to ensure ample soil water among treatments.

### Leaf gas exchange measurements

Leaf gas exchange was measured in September–October 2019 and 2021. Five healthy seedlings (i.e. no brown foliage, leaf curling, or wilting) were randomly selected per species per treatment and measured using a portable gas exchange system (LI-6400XT and LI-6800, LI-COR Biosciences Inc., Nebraska, United States of America). The LI-6400XT and LI-6800 were first cross-checked before the measurements to ensure they provided similar estimates of Anet across different air CO_2_ concentrations on the same leaf. Photosynthetic capacity (*V*_cmax_ and *J*_max_) was estimated using light-saturated CO_2_-response curves: the most recent, fully expanded leaves on each seedling were exposed to saturating light (1800 *µ*mol photons m^−2^ s^−1^) during which net CO_2_ assimilation rate (*A*_net_) was measured at varying concentrations of intercellular CO_2_ (*C*_i_). The first measurement was made at 400 *µ*mol CO_2_ mol^−1^ followed by a stepped sequence of 300, 200, 150, 100, 50, 400, 600, 800, 1000, 1,500, and 2000 *µ*mol CO_2_ mol^−1^. To characterize the relationship between photosynthetic capacity and short-term changes in temperature, these CO_2_-response curves were measured by placing the plant and gas exchange system inside temperature-controlled walk-in chambers (Environmental Growth Chambers, Ohio, United States of America). We then controlled the leaf temperature in the measuring cuvette at 10, 20, 30, or 40 °C for each response curve, and set the walk-in chamber to a similar temperature to ensure the gas exchange system and plant canopy experienced comparable thermal conditions as the measured leaf. Relative humidity inside the leaf chamber was kept between 60–80% during measurements. Each seedling was exposed to the target temperature for around 30 min, or until *A*_net_ was stable, before measurements were made. *A*_net_ measured at the growth [CO_2_] (*A*_growth_), *g*_s_, and the ratio of *C*_i_ to ambient [CO_2_] (*C*_i_/*C*_a_) were also recorded at each measurement temperature.

### Calculating *V*_cmax_ and *J*_max_

The C_3_ model of photosynthesis by Farquhar et al. (1980) was used to estimate *V*_cmax_ and *J*_max_ from the CO_2_-response data. Mesophyll conductance was not measured, so *C*_i_ was used (instead of the [CO_2_] at the site of carboxylation) to calculate an apparent *V*_cmax_ and apparent *J*_max_. Given the lack of information available on cuticular conductance in these species (and the lack of information on its acclimation to temperature and CO_2_ concentrations), we did not correct *C*_i_ for potential cuticular conductance effects (Márquez et al. 2021). However, we conducted a sensitivity analysis to explore the potential impact of accounting for cuticular conductance in our data, using the lowest (5 mmol m^−2^ s^−1^) and highest (20 mmol m^−2^ s^−1^) reported cuticular conductance values from Márquez et al. (2021). This analysis was performed only on black spruce, which has a very low stomatal conductance (making it highly sensitive to variation in potential cuticular conductance), and was limited to control plants (0TAC) at a leaf temperature of 20 °C. The sensitivity analysis showed that changes in cuticular conductance within this range do not affect *V_cmax_* and *J_max_* values (Supplementary Fig. S6).

Apparent *V*_cmax_ (µmol CO_2_ m^−2^ s^−1^) was calculated using:


(1) $$A_{c}=\frac{V_{cmax}(C_{i}-\Gamma^{*})}{C_{i}+K_{c}\left(1+\frac{O}{K_{o}}\right)}-R_{L}$$

where *A*_c_ is *A*_net_ when Rubisco activity is limiting (µmol CO_2_ m^−2^ s^−1^); *C*_i_ and *O* are the intercellular CO_2_ and O_2_ concentrations, respectively (µmol mol^−1^); *K*_c_ and *K*_o_ are the Michaelis-Menten coefficients of Rubisco's carboxylation and oxygenation reactions, respectively (µmol mol^−1^); $\Gamma^{*}$ is the CO_2_ compensation point in the absence of mitochondrial respiration (µmol CO_2_ mol^−1^); and *R*_L_ is respiration in the light (µmol CO_2_ m^−2^ s^−1^) (von Caemmerer 2000). Since there are no published values for *K*_c_, *K*_o_, $\Gamma^{*}$ or *R*_L_ in the full range of species studied here, we used values for tobacco (*Nicotiana tabacum*, L.), a species for which the photosynthetic response to [CO_2_] has been well-studied (Bernacchi et al. 2001), with standard temperature sensitivities from Medlyn et al. (2002). We also performed a sensitivity analysis to determine if the use of tobacco Rubisco kinetics affected our results, using diverse Rubisco kinetics from *Solanum tuberosum* and *Oryza sativa* in the msuRACIfit R package (McClain et al. 2025).

Apparent *J*_max_ (µmol CO_2_ m^−2^ s^−1^) was calculated from electron transport rates (*J*) at high CO_2_ concentrations using:


(2) $$\theta J^{2}-J(\alpha Q+J_{max})+\alpha QJ_{max}=0$$

(3) $$A_{j}=\frac{J}{4}\left(\frac{C_{i}-\Gamma^{*}}{C_{i}+2\Gamma^{*}}\right)-R_{L}$$

where *θ* is the curvature of the light–response curve (mol electrons mol^−1^ photons); *α* is the quantum yield of electron transport (mol electrons mol^−1^ photons); *Q* is the photosynthetic photon flux density (µmol photons m^−2^ s^−1^); and *A*_j_ is *A*_net_ when RuBP-regeneration is limiting (µmol CO_2_ m^−2^ s^−1^). The values for *θ* and *α* were fixed at 0.85 and 0.24 mol electrons mol^−1^ photons (similar to observed values for conifers: Medlyn et al. 2005).

The CO_2_-response curves for estimating apparent *V*_cmax_ and *J*_max_ were fitted using non-linear least square regressions via the fitacis() function from the *plantecophys* package (v. 1.4-6; Duursma 2015) in R (v. 4.2.1; R Core Team 2022). In addition to visually inspecting the curves and removing any with poor fits, curves with negative *C*_i_ values or that estimated unreasonable *V*_cmax_ or *J*_max_ values (e.g. below 10 *µ*mol CO_2_ m^−2^ s^−1^ or above 500 *µ*mol CO_2_ m^−2^ s^−1^) were also removed (a total of 31 out of 1,132 curves, or 2.7% of the total dataset).

### Modelling the short-term temperature responses of *A*_growth_, *V*_cmax_ and *J*_max_

The short-term temperature response of *A*_net_ measured at growth [CO_2_] (*A*_growth_; µmol CO_2_ m^−2^ s^−1^) was modelled using a quadratic regression (Kroner and Way 2016):


(4) $$A_{\mathrm{growth}}(T_{l})=aT_{l}^{2}+bT_{l}+c$$

where *A*_growth_ is *A*_net_ measured at 400 *µ*mol CO_2_ mol^−1^ for AC plants and 800 *µ*mol CO_2_ mol^−1^ for EC plants (the closest point to the 750 growth CO_2_  *µ*mol CO_2_ mol^−1^ concentration), *T*_l_ is the leaf temperature (°C), and *a*, *b*, and *c* are fitted constants. The thermal optimum of *A*_growth_ (*T*_optA_) was calculated by setting the derivative of Equation 4 equal to zero, then solving for *T*_l_; the maximum rate of *A*_growth_ (*A*_opt_, µmol CO_2_ m^−2^ s^−1^) is the rate of *A*_growth_ at *T*_optA_.

The short-term temperature responses of *V*_cmax_ and *J*_max_ were modelled using a peaked Arrhenius function (Kumarathunge et al. 2019; Medlyn et al. 2002):


(5) $$f(T_{l})=k_{\mathrm{opt}}\frac{H_{d}\exp\left(\frac{E_{a}(T_{l}-T_{\mathrm{opt}})}{T_{l}RT_{\mathrm{opt}}}\right)}{H_{d}-E_{a}\left(1-\exp\left(\frac{H_{d}(T_{l}-T_{\mathrm{opt}})}{T_{l}RT_{\mathrm{opt}}}\right)\right)}$$

where *k*_opt_ is the process rate (either *V*_cmax_ or *J*_max_) at its maximum value (*V*_cmaxopt_ or *J*_maxopt_, µmol CO_2_ m^−2^ s^−1^); *T*_opt_ is the thermal optimum of the process rate (*T*_optV_ for *V*_cmax_ and *T*_optJ_ for *J*_max_, degrees Kelvin); *T*_l_ is the leaf temperature (degrees Kelvin); *E*_a_ and *H*_d_ are the activation and deactivation energy terms that describe the increase in enzymatic activity below *T*_opt_ and the decrease in enzyme activity above *T*_opt_, respectively (*E*_aV_ for *V*_cmax_ and *E*_aJ_ for *J*_max_, J mol^−1^); and *R* is the universal gas constant (8.314 J mol^−1^ K^−1^). *H*_d_ was fixed at 200,000 J mol^−1^ to avoid over-parameterization (Medlyn et al. 2002; Kattge and Knorr 2007)—all other parameters were derived from the fitted curves. The temperature response curves for *A*_growth_, *V*_cmax_, and *J*_max_ were fitted in Microsoft Excel (v. 16.43; Microsoft 2020) following Equations 4 and 5.

### Leaf trait measurements

After measuring gas exchange, leaves were photographed, and projected leaf area was estimated using ImageJ (Abràmoff et al. 2004). Leaves were then dried at 60 °C to constant mass and weighed to calculate leaf mass per unit area (LMA, g m^−2^). The dry leaf tissue was ground into a fine powder with a Wiley Mill (Thomas Scientific, New Jersey, United States of America) and analysed for nitrogen concentration using an elemental analyser (vario ISOTOPE cube, Elementar, Germany).

### Statistical analyses

Data exploration was performed following the protocol described by Zuur et al. (2010). Linear mixed models were fitted for each species individually using the *nlme* package (v. 3.1-157; Pinheiro and Bates 2000), then repeated-measures analysis of variance (ANOVA) was applied to determine the effects of measurement year, leaf temperature, growth temperature, and growth [CO_2_] on *A*_growth_, *g*_s_, *C*_i_/*C*_a_, *V*_cmax_, and *J*_max_, with individual trees as random intercepts. Linear models and ANOVA were used to determine the effects of measurement year, growth temperature, and growth [CO_2_] on short-term temperature response parameters for *A*_growth_, *V*_cmax_, and *J*_max_ (i.e. *T*_optA_, *A*_opt_, and the parameters in Equation 5), as well as LMA and leaf nitrogen concentrations per unit leaf area (*N*_a_). Selection of fixed effects for all models was performed using Akaike's Information Criterion for small sample sizes (AICc). Tukey *post-hoc* tests with a significance value of 0.05 were performed on ANOVAs with a significant growth temperature effect or interaction effects to compare across treatments. All data are presented as means ± standard error unless otherwise stated.

## Supplementary Material

kiaf380_Supplementary_Data

## Data Availability

Data available on request.

## Supplementary data

The following materials are available in the online version of this article.

**Supplementary Figure S1.** Short-term response to leaf temperature of stomatal conductance to water vapor (*g*_s_).

**Supplementary Figure S2.** Short-term response to leaf temperature of the ratio of intercellular [CO_2_] to ambient [CO_2_] (*C*_i_/*C*_a_).

**Supplementary Figure S3.** Short-term response to leaf temperature of maximum rate of Rubisco carboxylation (*V*_cmax_) in black spruce (*Picea mariana*).

**Supplementary Figure S4.** Leaf nitrogen per unit area (*N*_a_).

**Supplementary Figure S5.** Leaf mass per unit area (LMA).

**Supplementary Figure S6.** The potential impact of cuticular conductance variation on photosynthetic capacity estimates.

**Supplementary Table S1.** Summary of two-way ANOVA results for short-term temperature response parameters and leaf traits, analyzed within measurement year.

## Dive Curated Terms

The following phenotypic, genotypic, and functional terms are of significance to the work described in this paper:

leaf temperature Planteome: TO:0000504

stomatal conductance Planteome: TO:0000522

triose CHEBI: CHEBI:27137
