# Supplementary material for: Photosynthetic capacity is reduced by warming but unaffected by elevated CO2 in seedlings of five boreal tree species
Source: Plant Physiol. 2025 Aug 29;199(1):kiaf380. doi: 10.1093/plphys/kiaf380 (PMC12448708; doi:10.1093/plphys/kiaf380)
Supplement: kiaf380_Supplementary_Data [file kiaf380_supplementary_data.docx]

**Photosynthetic capacity is reduced by warming but unaffected by elevated CO_2_ in seedlings of five boreal tree species**

Julia M. Hammer^1*^, Mirindi Eric Dusenge^1,2,3,4^, Nick Bither^5,6^, Andrew Cook^1^, André G. Duarte^1^, Kiana Lee^1^, Bridget K. Murphy^1,7,8^, Melissa A. Pastore^9,10^, Stephanie C. Schmiege^1,11^, Robyn Swartman^1^, Raimundo Bermudez^5^, Norman P. A. Hüner^1^, Peter B. Reich^5,12,13^, Danielle A. Way^1,4,14,15*^

^1^ Department of Biology, University of Western Ontario, London, ON, Canada

^2^ Western Centre for Climate Change, Sustainable Livelihoods and Health, Department of Geography, University of Western Ontario, London, ON, Canada

^3^ Department of Biology, Mount Allison University, Sackville, NB, Canada

^4^ Research School of Biology, Australian National University, Acton, ACT, Australia

^5^ Department of Forest Resources, University of Minnesota, Saint Paul, MN, USA

^6^ Department of Biological Sciences, University of Denver, Denver, CO, USA

^7^ Department of Biology, University of Toronto Mississauga, Mississauga, ON, Canada

^8^ Graduate program in Cell and Systems Biology, University of Toronto, Toronto, ON, Canada

^9^ Department of Ecology, Evolution, and Behavior, University of Minnesota, Saint Paul, MN, USA

^10^ USDA Forest Service, Northern Research Station, St. Paul, MN, USA

^11^ Plant Resilience Institute, Michigan State University, East Lansing, MI, USA

^12^ Hawkesbury Institute for the Environment, Western Sydney University, Penrith, NSW, Australia

^13^ Institute for Global Change Biology and School for the Environment and Sustainability, University of Michigan, Ann Arbor, MI, USA

^14^ Environmental & Climate Sciences Department, Brookhaven National Laboratory, Upton, NY, USA

^15^ Nicholas School of the Environment, Duke University, Durham, NC, USA

*Contact authors: [jhammer2@uwo.ca](mailto:jhammer2@uwo.ca), [danielle.way@anu.edu.au](mailto:danielle.way@anu.edu.au)

**Supplementary Figure S1.** Short-term response to leaf temperature of stomatal conductance to water vapor (*g*_s_). Data from five boreal tree species grown under either ambient or elevated [CO_2_], and either ambient temperatures (0T, grey circles), ambient +4 ºC (4T, yellow squares), or ambient +8 ºC (8T, red diamonds), in 2019 (left) and 2021 (right). Repeated measures ANOVAs were performed for each year-species combination: main effects were leaf temperature (*T*_l_), growth temperature (*T*_g_), and CO_2_ environment, with individual tree-*T*_l_ relationships as random effects. Means $\boldsymbol{\pm}$ SE, n = 3-7 (except in d, where n = 2-6). **p*<0.05, ***p*<0.01, ****p*<0.001.

**Supplementary Figure S2.** Short-term response to leaf temperature of the ratio of intercellular [CO_2_] to ambient [CO_2_] (*C*_i_/*C*_a_). Data from five boreal tree species grown under either ambient or elevated [CO_2_], and either ambient temperatures (0T, grey circles), ambient +4 ºC (4T, yellow squares), or ambient +8 ºC (8T, red diamonds), in 2019 (left) and 2021 (right). Repeated measures ANOVAs were performed for each year-species combination: main effects were leaf temperature (*T*_l_), growth temperature (*T*_g_), and CO_2_ environment, with individual tree-*T*_l_ relationships as random effects. Means $\boldsymbol{\pm}$ SE, n = 3-7 (except in d, where n = 2-6). **p*<0.05, ***p*<0.01, ****p*<0.001.

**Supplementary Figure S3.** Short-term response to leaf temperature of maximum rate of Rubisco carboxylation (*V*_cmax_) in black spruce (*Picea mariana*). Data from black spruce grown under either ambient (AC, left) or elevated [CO_2_] (EC, right), and either ambient temperatures (0T, grey circles), ambient +4 ºC (4T, yellow circles), or ambient +8 ºC (8T, red circles). Tobacco, *V*_cmax_ calculated assuming tobacco Rubisco kinetics as shown in main text; S_tuberosum, *V*_cmax_ calculated assuming *Solanum tuberosum* Rubisco kinetics; O_Sativa, *V*_cmax_ calculated assuming *Oryza sativa* Rubisco kinetics. Means $\boldsymbol{\pm}$ SE, n = 5.

**Supplementary Figure S4.** Leaf nitrogen per unit area (*N*_a_). Data from five boreal tree species grown under either ambient (AC) or elevated CO_2_ (EC), and either ambient temperatures (0T, grey circles), ambient +4 ºC (4T, yellow squares), or ambient +8 ºC (8T, red diamonds) in 2019 and 2021. Three-way ANOVAs were performed on each parameter for each species, with measurement year (Yr), growth temperature (*T*_g_) and CO_2_ environment as main effects. Measurement year did not improve model fit in paper birch (e), so data were combined across years and two-way ANOVAs with only *T*_g_ and CO_2_ as main effects were performed. Letters represent post-hoc Tukey comparisons across the six treatments at p<0.05. Means $\boldsymbol{\pm}$ SE, n = 3-7 (except in a and d, where n = 1-6). **p*<0.05, ***p*<0.01, ****p*<0.001.

**Supplementary Figure S5.** Leaf mass per unit area (LMA). Data from five boreal tree species grown under either ambient (AC) or elevated CO_2_ (EC), and either ambient temperatures (0T, grey circles), ambient +4 ºC (4T, yellow squares), or ambient +8 ºC (8T, red diamonds) in 2019 and 2021. Three-way ANOVAs were performed on each parameter for each species, with measurement year (Yr), growth temperature (*T*_g_) and CO_2_ environment as main effects. Letters represent post-hoc Tukey comparisons across the six treatments at p<0.05. Means $\boldsymbol{\pm}$ SE, n = 3-7 (except in a and e, where n = 1-6). **p*<0.05, ***p*<0.01, ****p*<0.001.

**Supplementary Figure S6.** The potential impact of cuticular conductance variation on photosynthetic capacity estimates. Impact of considering potential cuticular conductance variation on a-b) the maximum rate of Rubisco carboxylation (*V*_cmax_); and c-d) the maximum rate of electron transport (*J*_max_) in black spruce (*Picea mariana*) grown under ambient [CO_2_] and ambient temperatures in 2019 (left) and 2021 (right). Vcmax_Cut_20, *V*_cmax_ calculated assuming a cuticular conductance of 20 mmol m^-2^ s^-1^; Vcmax_Cut_5, *V*_cmax_ calculated assuming a cuticular conductance of 5 mmol m^-2^ s^-1^; Vcmax_ob, *V*_cmax_ calculated assuming cuticular conductance is 0 mmol m^-2^ s^-1^ (as reported in the main text). Means $\boldsymbol{\pm}$ SE, n = 5. There were no significant differences between the three cuticular conductance estimates for any figure based on a one-way ANOVA at p<0.05.

**Supplementary Table S1.** Summary of two-way ANOVA results for short-term temperature response parameters and leaf traits, analyzed within measurement year. Only *p*-values shown. Main effects were growth temperature (*T*_g_) and growth [CO_2_]. Empty cells represent interaction effects that did not contribute to model fit (estimated by AICc; see Methods). Traits analyzed were the maximum rate of *A*_growth_ (*A*_opt_, µmol CO_2_ m^-2^ s^-1^), the thermal optimum of *A*_growth_ (*T*_optA_, $^{\circ}$C), the maximum rates of *V*_cmax_ (*V*_cmaxopt_, µmol CO_2_ m^-2^ s^-1^), and *J*_max_ (*J*_maxopt_, µmol CO_2_ m^-2^ s^-1^), the thermal optimums of *V*_cmax_ (*T*_optV_, $^{\circ}$C) and *J*_max_ (*T*_optJ_, $^{\circ}$C), the activation energies of *V*_cmax_ (*E*_aV_, J mol^-1^) and *J*_max_ (*E*_aJ_, J mol^-1^), *V*_cmax_ and *J*_max_ measured at 20 $^{\circ}$C (*V*_cmax20_ and *J*_max20_, µmol CO_2_ m^-2^ s^-1^), the ratio of *J*_max20_ to *V*_cmax20_ (*J*_max20_/*V*_cmax20_), leaf nitrogen per unit area (*N*_a_, g m^-2^), and leaf mass per unit area (LMA, g m^-2^). Numbers in bold and italics represent *p*<0.05 and 0.05<*p*<0.1, respectively.

**Supplementary Figure S1**


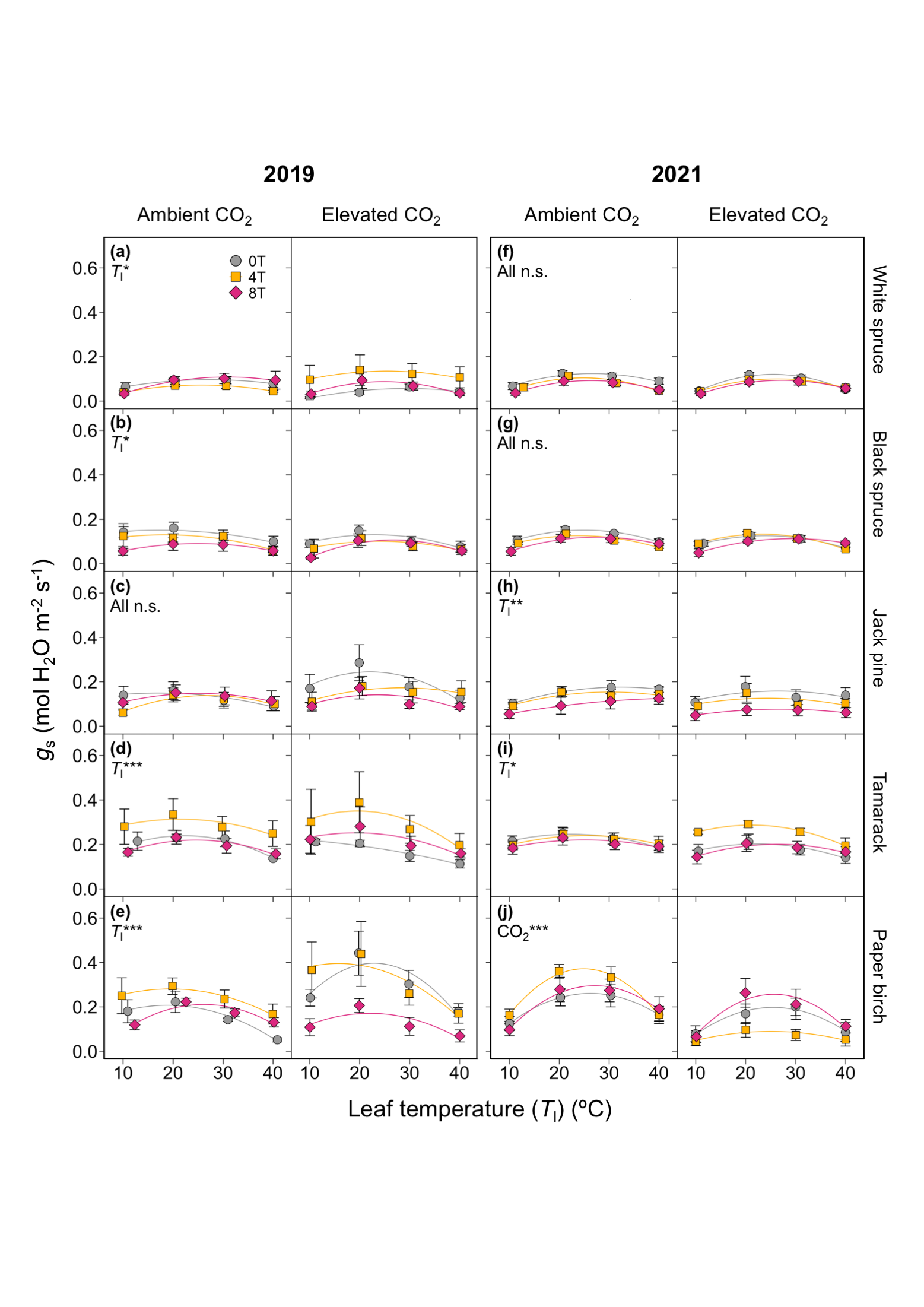


**Supplementary Figure S2**


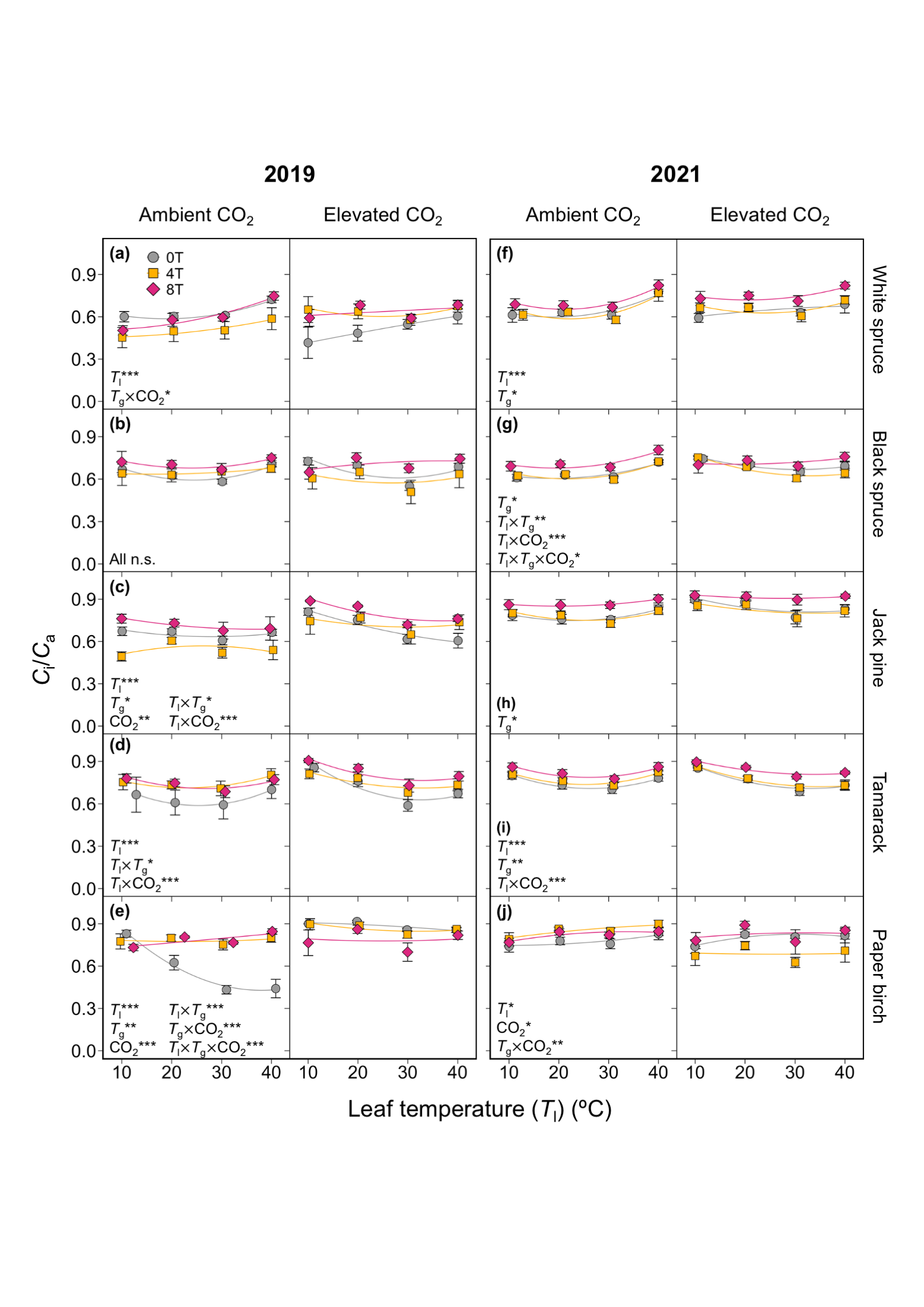


**Supplementary Figure S3**

**
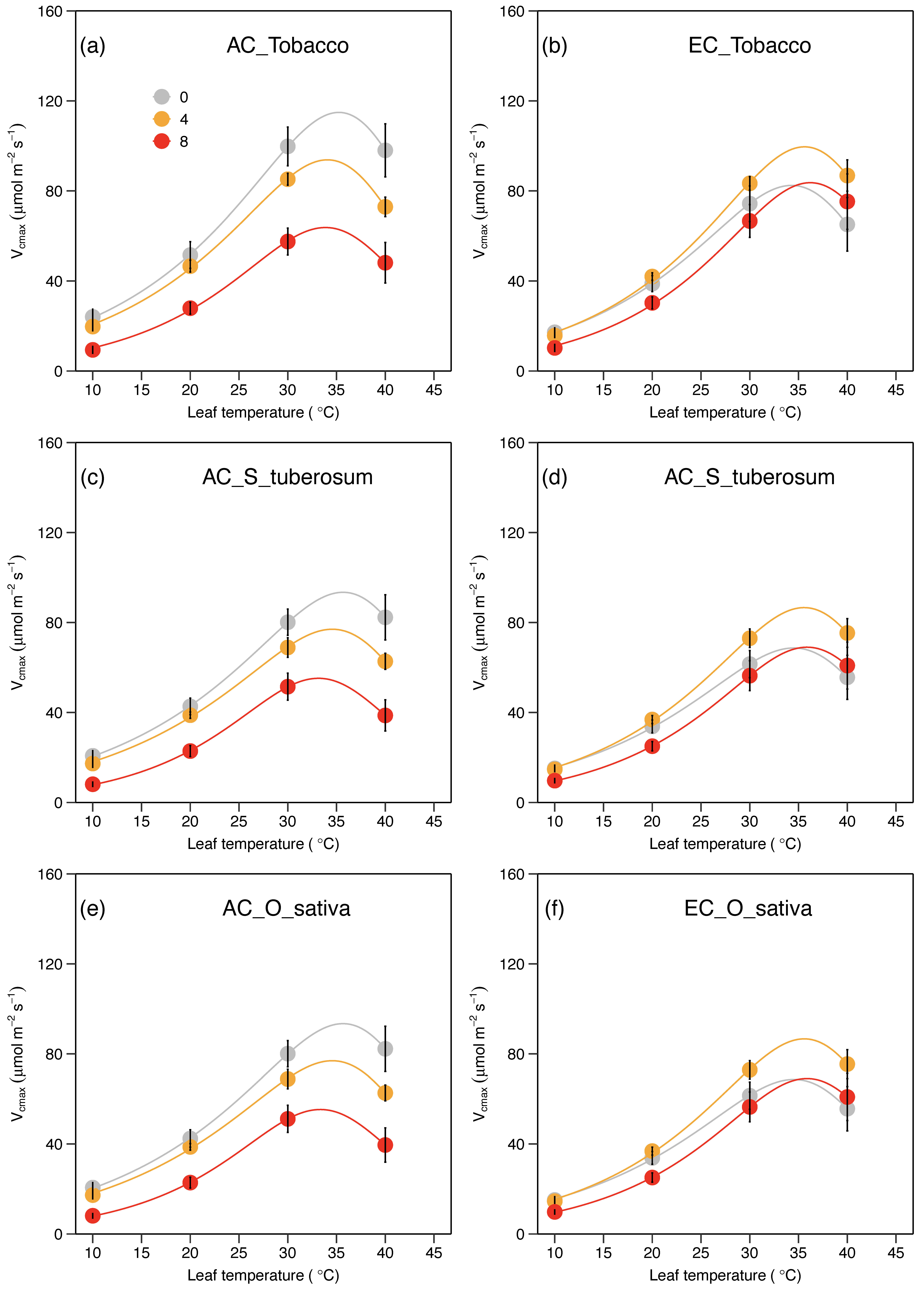
**

**Supplementary Figure S4**


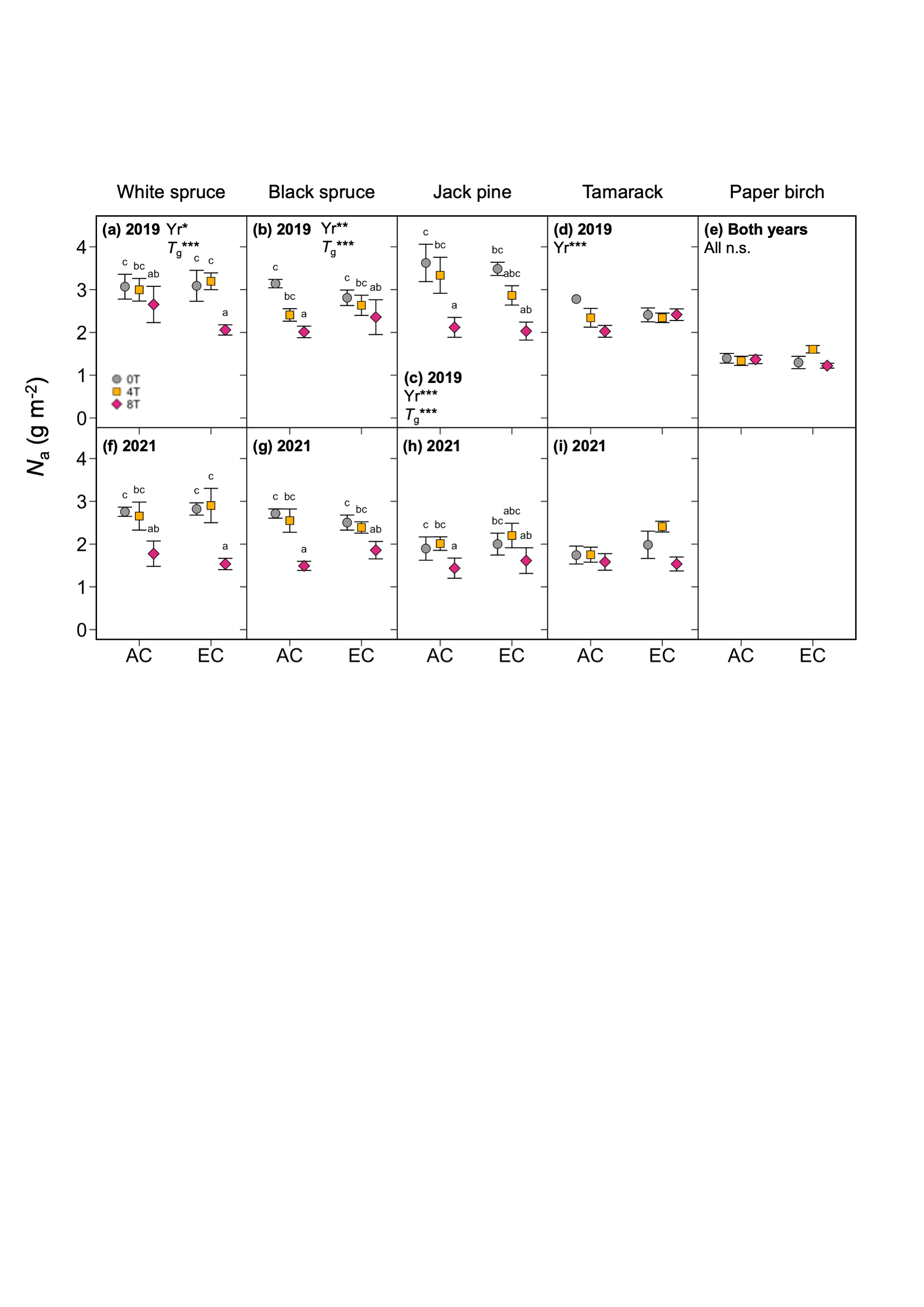


**Supplementary Figure S5**


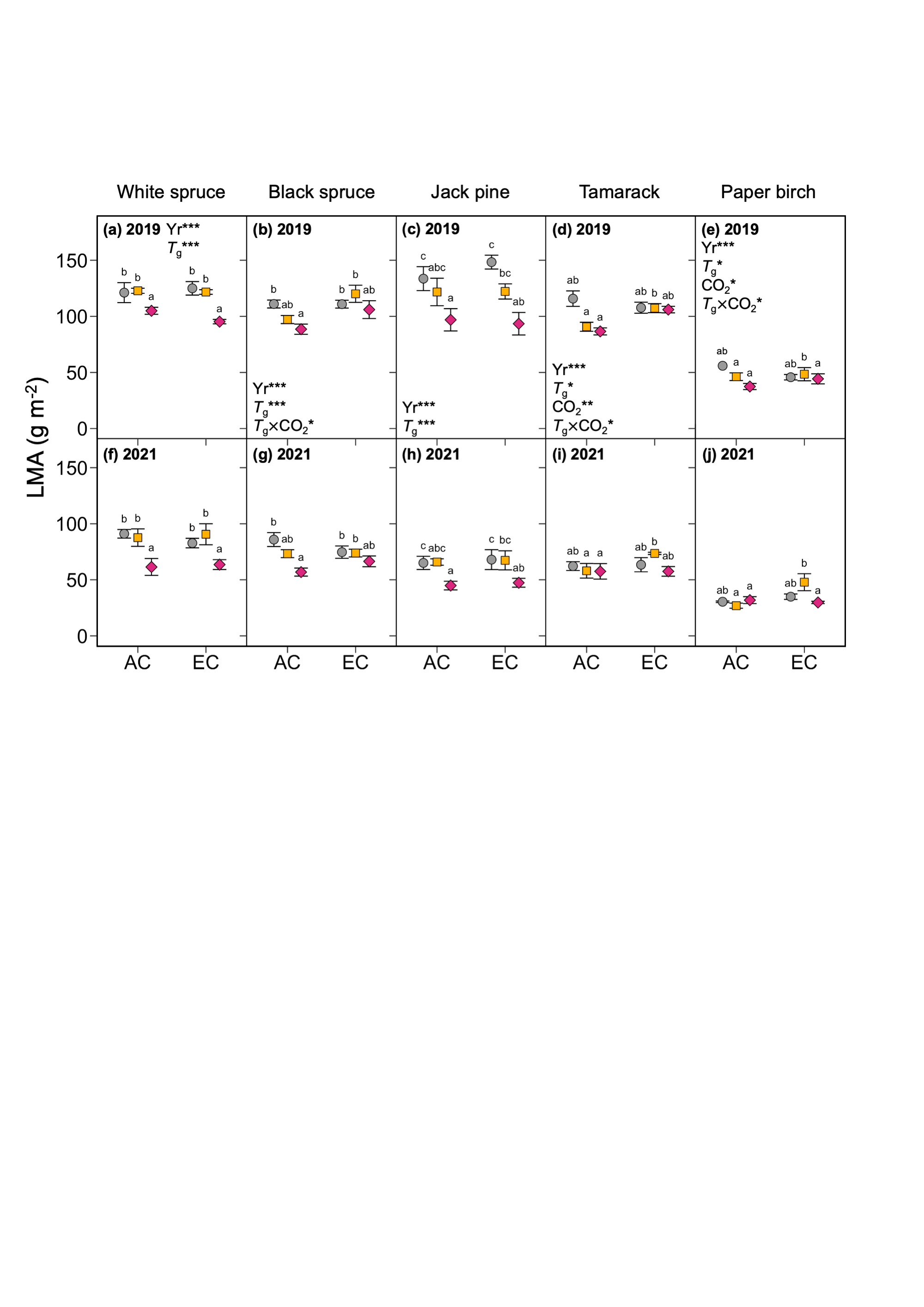


**Supplementary Figure S6**


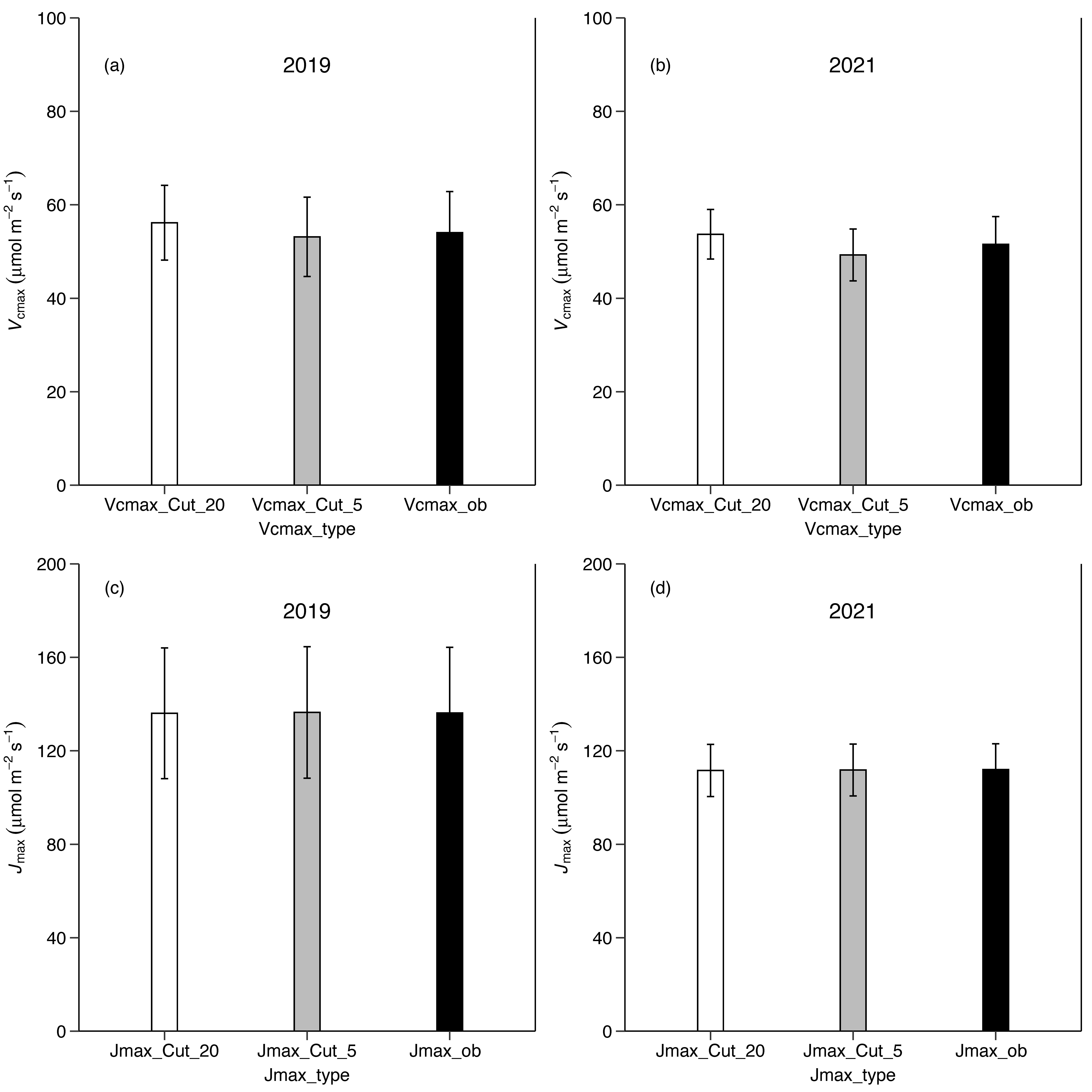


**Supplementary Table S1**

| **Parameter** | **White spruce** | | **Black spruce** | | **Jack pine** | | **Tamarack** | | **Paper birch** | |
| --- | --- | --- | --- | --- | --- | --- | --- | --- | --- | --- |
| **Effect** | **2019** | **2021** | **2019** | **2021** | **2019** | **2021** | **2019** | **2021** | **2019** | **2021** |
| ***A*_opt_** |  |  |  |  |  |  |  |  |  |  |
| ***T*_g_** | 0.37 | **0.001** | **0.02** | **<.0001** | **0.01** | **0.048** | **0.01** | **0.01** | **0.0005** | 0.15 |
| **CO_2_** | **0.02** | **<.0001** | **0.0007** | **<.0001** | **0.01** | 0.24 | **0.008** | **<.0001** | 0.5 | 0.14 |
| ***T*_g_**$\boldsymbol{\times}$ **CO_2_** |  |  |  |  | *0.06* |  |  | *0.07* | **0.02** |  |
| ***T*_optA_** |  |  |  |  |  |  |  |  |  |  |
| ***T*_g_** | 0.89 | *0.07* | **0.03** | **<.0001** | **0.04** | 0.99 | *0.09* | **0.002** | *0.06* | 0.34 |
| **CO_2_** | **0.009** | **0.006** | **0.02** | **<.0001** | **0.0009** | **0.04** | **0.002** | **<.0001** | 0.77 | 0.11 |
| ***T*_g_**$\boldsymbol{\times}$ **CO_2_** | **0.03** |  |  |  |  |  |  |  |  |  |
| ***V*_cmaxopt_** |  |  |  |  |  |  |  |  |  |  |
| ***T*_g_** | 0.41 | **0.001** | **0.03** | **0.01** | **0.03** | **0.04** | 0.34 | **0.02** | **<.0001** | 0.77 |
| **CO_2_** | 0.57 | 0.99 | 0.55 | 0.91 | 0.77 | 0.27 | 0.40 | 0.15 | **<.0001** | *0.07* |
| ***T*_g_**$\boldsymbol{\times}$ **CO_2_** |  |  |  | **0.01** |  |  |  |  | **<.0001** |  |
| ***J*_maxopt_** |  |  |  |  |  |  |  |  |  |  |
| **Yr** | 0.62 | **0.002** | **0.04** | **0.0005** | **0.03** | *0.06* | 0.12 | **0.02** | **0.0007** | 0.10 |
| ***T*_g_** | 0.98 | 0.92 | 0.57 | 0.74 | 0.37 | 0.59 | 0.85 | 0.30 | **0.002** | **0.02** |
| **CO_2_** |  |  |  | **0.04** |  |  |  |  | **0.03** |  |
| ***T*_optV_** |  |  |  |  |  |  |  |  |  |  |
| ***T*_g_** | *0.09* | 0.29 | 0.39 | 0.93 | 0.06 | 0.80 | 0.39 | 0.53 | 0.79 | 0.29 |
| **CO_2_** | 0.34 | 0.63 | 0.65 | *0.09* | 0.13 | 0.73 | 0.71 | 0.21 | 0.28 | 0.77 |
| ***T*_g_**$\boldsymbol{\times}$ **CO_2_** |  |  |  | *0.07* |  |  |  |  |  |  |
| ***T*_optJ_** |  |  |  |  |  |  |  |  |  |  |
| ***T*_g_** | **0.03** | 0.35 | 0.14 | 0.48 | 0.31 | 0.15 | 0.12 | 0.69 | 0.94 | 0.37 |
| **CO_2_** | 0.27 | 0.69 | 0.62 | 0.17 | 0.27 | 0.97 | 0.46 | 0.31 | 0.74 | 0.83 |
| ***T*_g_**$\boldsymbol{\times}$ **CO_2_** |  | **0.004** |  |  |  |  |  |  |  |  |
| ***E*_aV_** |  |  |  |  |  |  |  |  |  |  |
| ***T*_g_** | 0.82 | **0.02** | 0.55 | *0.07* | **0.02** | 0.64 | 0.21 | *0.07* | 0.60 | 0.13 |
| **CO_2_** | 0.53 | 0.83 | 0.23 | 0.94 | 0.20 | 0.92 | 0.92 | 0.90 | 0.40 | 0.32 |
| ***T*_g_**$\boldsymbol{\times}$ **CO_2_** |  |  |  |  |  |  |  |  | **0.01** |  |
| ***E*_aJ_** |  |  |  |  |  |  |  |  |  |  |
| ***T*_g_** | 0.84 | **0.01** | 0.33 | **0.006** | 0.42 | 0.48 | 0.58 | **0.046** | 0.27 | 0.28 |
| **CO_2_** | 0.22 | 0.23 | 0.71 | 0.73 | 0.46 | 0.97 | 0.75 | 0.28 | 0.29 | 0.35 |
| ***T*_g_**$\boldsymbol{\times}$ **CO_2_** |  |  |  |  |  |  |  |  | **0.004** |  |
| ***J*_max20_/*V*_cmax20_** |  |  |  |  |  |  |  |  |  |  |
| ***T*_g_** | 0.98 | 0.27 | 0.27 | 0.35 | 0.17 | 0.12 | 0.57 | 0.27 | **0.003** | 0.19 |
| **CO_2_** | 0.17 | 0.38 | 0.48 | 0.19 | *0.08* | 0.38 | **0.03** | **0.003** | **0.0001** | **0.03** |
| ***T*_g_**$\boldsymbol{\times}$ **CO_2_** | **0.002** |  |  |  |  |  |  |  | **0.008** |  |
| ***N*_a_** |  |  |  |  |  |  |  |  |  |  |
| ***T*_g_** | **0.007** | **0.0001** | **0.002** | **<.0001** | **0.0002** | 0.12 | 0.41 | *0.07* | 0.23 | 0.95 |
| **CO_2_** | 0.58 | 0.90 | 0.80 | 0.99 | 0.32 | 0.46 | 0.46 | 0.10 | 0.38 | 0.24 |
| ***T*_g_**$\boldsymbol{\times}$ **CO_2_** |  |  |  |  |  |  |  |  |  | **0.04** |
| **LMA** |  |  |  |  |  |  |  |  |  |  |
| ***T*_g_** | **0.0001** | **0.0003** | **0.02** | **0.003** | **0.0002** | **0.02** | **0.007** | 0.30 | *0.08* | 0.29 |
| **CO_2_** | 0.65 | 0.82 | **0.006** | 0.93 | 0.59 | 0.70 | **0.008** | 0.19 | 0.69 | **0.009** |
| ***T*_g_**$\boldsymbol{\times}$ **CO_2_** |  |  | *0.06* |  |  |  | **0.009** |  |  | **0.009** |
